# Micro RNAs of Epstein-Barr Virus Promote Cell Cycle Progression and Prevent Apoptosis of Primary Human B Cells

**DOI:** 10.1371/journal.ppat.1001063

**Published:** 2010-08-19

**Authors:** Eri Seto, Andreas Moosmann, Sebastian Grömminger, Nicole Walz, Adam Grundhoff, Wolfgang Hammerschmidt

**Affiliations:** 1 Department of Gene Vectors, Helmholtz Zentrum München, German Research Center for Environmental Health, Munich, Germany; 2 Clinical Cooperation Group Molecular Oncology, Ludwig Maximilians-University Munich and Helmholtz Zentrum München, German Research Center for Environment and Health, Munich, Germany; 3 Institute for Clinical and Molecular Biology, Helmholtz Zentrum München, German Research Center for Environment and Health, Munich, Germany; 4 Heinrich-Pette-Institute for Experimental Virology and Immunology, Hamburg, Germany; Emory University, United States of America

## Abstract

Cellular and viral microRNAs (miRNAs) are involved in many different processes of key importance and more than 10,000 miRNAs have been identified so far. In general, relatively little is known about their biological functions in mammalian cells because their phenotypic effects are often mild and many of their targets still await identification. The recent discovery that Epstein-Barr virus (EBV) and other herpesviruses produce their own, barely conserved sets of miRNAs suggests that these viruses usurp the host RNA silencing machinery to their advantage in contrast to the antiviral roles of RNA silencing in plants and insects. We have systematically introduced mutations in EBV's precursor miRNA transcripts to prevent their subsequent processing into mature viral miRNAs. Phenotypic analyses of these mutant derivatives of EBV revealed that the viral miRNAs of the *BHRF1* locus inhibit apoptosis and favor cell cycle progression and proliferation during the early phase of infected human primary B cells. Our findings also indicate that EBV's miRNAs are not needed to control the exit from latency. The phenotypes of viral miRNAs uncovered by this genetic analysis indicate that they contribute to EBV-associated cellular transformation rather than regulate viral genes of EBV's lytic phase.

## Author Summary

Micro RNAs (miRNAs) are small, non-coding RNAs that bind to mRNA transcripts and abrogate their protein coding functions. Only a few of their mRNA targets are known, although miRNAs are found in all multicellular organisms and certain viruses. In particular, members of the herpesvirus family encode a surprisingly large number of miRNAs. Epstein-Barr virus (EBV) belongs to this virus family and is associated with several human malignancies including B-cell lymphomas. In vitro, this virus infects human primary B cells and transforms them into continuously proliferating lymphoblastoid cell lines (LCL), which is an amenable model covering key aspects of cellular transformation and lymphomagenesis. To assess the roles of EBV's miRNAs in this model, we generated EBV mutants that lack the capacity to encode viral miRNAs. Phenotypic analysis of human primary B cells infected with these mutant viruses revealed that miRNAs encoded in EBV's *BHRF1* locus strongly promote B cell proliferation, regulate cell cycle functions, and prevent apoptosis early after infection. Our findings show that EBV has evolved discrete miRNAs to contribute to its well-known transforming capacity, which has not been appreciated previously.

## Introduction

Thousands of microRNAs (miRNAs) have been identified so far (miRBase, release 14, Sept. 2009; http://www.mirbase.org), which are small noncoding single-stranded RNAs of about 21 to 25 nucleotides in length. They are found transcribed in all multicellular organisms and certain viruses and often are phylogenetically conserved across species [1]–[3]. The 5′-ends of miRNAs, the so-called seed sequences, recognize partially complementary mRNA targets usually within their 3′ untranslated regions and repress translational of these mRNAs [4]. In recent years, miRNAs have emerged as key regulators of a number of biological processes including developmental timing, differentiation and pattering, but also cellular proliferation, cell death, immune response, haematopoesis, and cellular transformation or oncogenesis [5]–[10]. Individual miRNAs can directly regulate the expression of hundreds of different mRNAs [11] and possibly influence the steady state levels of more than 30% of the proteins in mammalian cells [2], [12].

One standard approach to identify targets of miRNAs relies on computational algorithms that build on the thermodynamic stability of miRNA/mRNA complexes and the evolutionary conservation of miRNA seed sequences [13] because sequences of the (cellular) mRNA target molecules are frequently preserved across species [10]. One major disadvantage of this dual approach lies in a large number of false positive predictions because many putative mRNA target sites might not be accessible due to mRNA folding. In addition, as this computational approach eliminates potential targets that are not conserved between different species or related viruses, it is inadequate for predicting targets of herpesviral miRNAs because their evolutionary conservation is surprisingly low among members of the herpesvirus family [14]–[16].

An alternative approach uses microarray analyses of cellular mRNAs upon ectopic expression of individual or multiple miRNAs [5], [17], [18]. This approach is useful to reveal direct and indirect downstream targets of miRNAs but it may miss authentic targets if their mRNA levels are not sufficiently down-regulated for reliable detection by microarray analysis. In addition, antisense oligonucleotides [19], [20] or competitive inhibitors [21] have been used for the experimental identification and/or subsequent verifications of potential target genes.

The identification and functional assessment of miRNAs can reveal a rich biology. One prominent example is the human miR-155, the product of the *bic* gene [22]. miR-155 was found to be overexpressed in several types of B-cell lymphoma [23] and its transgenic expression in mice caused B-cell malignancies [24]. miR-155 is an orthologue of Kaposi sarcoma-associated herpesvirus (KSHV)-encoded miR-K12-11 [25], [26] and candidate target genes, identified by microarray analysis, were confirmed to be regulated similarly by miR-K12-11 [ibid and 17]. Beyond this prominent example, relatively few targets of viral miRNAs have been experimentally confirmed probably due to a large number of false positive predictions and poor evolutionary conservation of viral miRNAs [14].

We have generated recombinant EBVs modified in their capacity to encode EBV's miRNAs to probe their functions in the viral life cycle. We show that viral mutants deficient in BHRF1 miRNAs are dramatically reduced in their support of proliferation of infected B cells early after infection. B cell newly infected with EBV lacking the BHRF1 miRNAs progressed through the cell cycle less efficiently and died by apoptosis more often than cells infected identically with the parental EBV. Our phenotypic characterization revealed that EBV's miRNAs support EBV-mediated B-cell activation but play no apparent role in maintaining viral latency in contrast to the miRNAs of Kaposi's sarcoma-associated herpesvirus (KSHV) [27 and references therin] showing that the miRNAs of related human γ-herpesviruses evolved to perform divergent sets of functions.

## Results

### Functional deletion and reconstitution of viral miRNAs in B95.8-based mutant EBVs

**Figure 1:**
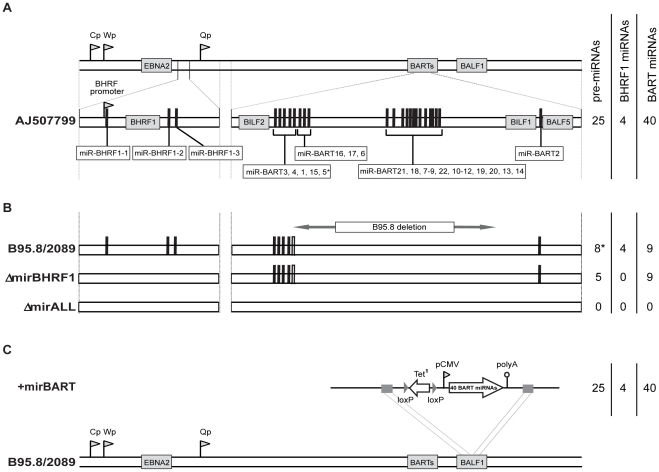
Schematic overview of the construction of miRNA-mutated EBVs. (A) Genomic localization of EBV's miRNAs in the EBV reference strain AJ507799. The two miRNA families of EBV originate from two transcripts that fold into 25 pre-miRNAs as indicated by black bars and give rise to four mature BHRF1 miRNAs and 40 BART miRNAs. (B) Functional ablation of EBV's miRNAs. Prototype 2089 EBV is based on the prototypic EBV strain B95.8 [29], which was cloned in *E. coli* [28]. As compared to the Genbank entry of the hypothetical EBV reference strain AJ507799, the genome of B95.8 suffers from a deletion and therefore lacks the coding capacity of the majority of the BART miRNAs as indicated. In *E. coli*, the three pre-miRNA structures in the BHRF1 locus of prototype 2089 EBV were replaced with computed scrambled sequences (Table 1) to generate a functional BHRF1 miRNA knock-out EBV termed ΔmirBHRF1 (or p4004). In a subsequent step, the remaining five BART pre-miRNAs were replaced with scrambled sequences to generate an EBV genome devoid of any miRNAs. This mutant was termed ΔmirALL (or 4027). The sequence of miR-BART5 is present in the genome of prototype 2089 EBV but due to the deletion in the parental B95.8 strain the pre-miRNA cannot form the hairpin structure needed for miR-BART5's processing by Drosha as indicated (*) in Figure 1B. (C) Construction of a reconstituted EBV mutant encoding 22 BART pre-miRNAs. The expression cassette p4079, which encodes all pre-miRNAs of the BART locus, was introduced into the *BALF1* locus of the prototype 2089 EBV genome by homologous recombination as indicated. The EBV mutant that carries all BART miRNAs encoded by conventional EBV strains, was termed +mirBART (or p4080).

We assessed the role of EBV's miRNAs in EBV-mediated B-cell activation, transformation and/or viral latency genetically. All EBV's miRNAs are clustered in two areas of the genome, the *BHRF1* and *BART* genes. We generated two recombinant EBV mutants that carry inactivated alleles of the BHRF1 or BART miRNAs or both (Figure 1) on the basis of the E.coli-cloned genome of the B95.8 strain of EBV [28]. This EBV genome, designated 2089 and regarded as the recombinant version of prototypic EBV [29], encodes three BHRF1 and five BART pre-miRNAs, which are processed to four BHRF1 and nine BART mature miRNAs, respectively (Figure 1B). To replace the wild-type alleles by nonfunctional alleles of the viral miRNAs, we altered all eight pre-miRNAs from which the 13 mature miRNAs sequences of this EBV strain arise to computed, scrambled versions that are expected to interfere with Drosha processing (Table 1 and Supporting Figure S1). All EBV miRNAs are located in non-coding regions of the BHRF1 and BART transcripts and genetic modifications within these sequences are therefore not expected to affect the protein coding capacities of both genes. The scrambled primary RNA sequences were designed to maintain the wild-type nucleotide composition and the overall genomic architecture of the original EBV DNA but to be unable to fold into the specific hairpin structures of pri-miRNAs. As a consequence the nuclear RNaseIII enzyme Drosha would not process the scrambled RNAs and no mature functional miRNAs could form. We replaced EBV's pre-miRNAs with their scrambled mutant sequences (Figure 1B and Supporting Figure S1) using *galK*-mediated recombination [30] in four consecutive rounds of genetic manipulation in *E. coli* (see Material and Methods for experimental details). The two final EBV mutants were checked by detailed restriction enzyme analyses. DNA sequencing confirmed the intended genetic alterations of the viral pre-miRNAs and the integrity of the maxi-EBV genomes. ΔmirBHRF1 EBV lacks the coding capacity of the BHRF1 miRNA locus and ΔmirALL EBV is devoid of all viral miRNAs (Figure 1B). Both mutant EBVs are otherwise prototype 2089 without any further genetic alterations, additional marker genes, or their remnants.

**Table 1:** Wild-type sequences and corresponding scrambled versions of EBV-encoded miRNAs.

miRNA	Wild-type sequence (5′ to 3′)	Scrambled sequence (5′ to 3′)
miR-BHRF1-1	UAACCUGAUCAGCCCCGGAGUU	GAUAAUAACCCGGGUGCCCCUU
miR-BHRF1-2-5p*	AAAUUCUGUUGCAGCAGAUAGC	CGGUAGGUGAUAUCCAAACUAU
miR-BHRF1-2-3p	UAUCUUUUGCGGCAGAAAUUGA	GAAACUUCUGGAUCGUGAUUAU
miR-BHRF1-3	UAACGGGAAGUGUGUAAGCACA	UGAAGAUAAGGUCCACGGAAUG
miR-BART3-5p*	ACCUAGUGUUAGUGUUGUGCU	GCCUAGUUUCGGUUGUGUAAU
miR-BART3-3p	CGCACCACUAGUCACCAGGUGU	UGUGUCCACCACAAACUGCCGG
miR-BART4	GACCUGAUGCUGCUGGUGUGCU	CAGAGCCUUUUGGGUCGGUCGU
miR-BART4*	CACAUCACGUAGGCACCAGGUGU	#)
miR-BART1-5p	UCUUAGUGGAAGUGACGUGCUGUG	UGGGACCUUAUGGUAUGUGGAUCG
miR-BART1-3p	UAGCACCGCUAUCCACUAUGUC	CAAUUAGAUUCACCCUCCCUGG
miR-BART15	GUCAGUGGUUUUGUUUCCUUGA	UCGCUUUGUUUUGUUGUGACAG
miR-BART5+)	CAAGGUGAAUAUAGCUGCCCAUCG	UUCAAUCAACGGAGUCGAAGUCCG
miR-BART2-5p	UAUUUUCUGCAUUCGCCCUUGC	CGAACUUCUUUCCGUGUUUUCC
miR-BART2-3p	AAGGAGCGAUUUGGAGAAAAUAAA	AAGAUGAAAAUCGAUUAGGGGAAA

# ) The sequence of miR-BART4* is present in all 2089-derived mutant EBVs but its pre-miRNA cannot form the necessary stem of the hairpin structure in ΔmirALL because the sequence of the 5′ arm is scrambled as in miR-BART4.

**+:** ) The sequence of miR-BART5 is present in prototype 2089 EBV but due to the deletion in the parental B95.8 EBV strain it cannot fold into the pre-miRNA hairpin structure and is therefore not processed to the mature miR-BART5 even in the prototype 2089 EBV and B95.8 strains.

As shown in Figure 1A, EBV field strains other than the reference strain B95.8 encode up to 25 pre-miRNAs, which result in four mature BHRF1 miRNAs and 40 BART miRNAs. To examine the role of BART miRNAs that are not encoded in B95.8, we generated the reconstituted EBV mutant that ectopically expresses the full set of all BART miRNAs (+mirBART in Figure 1C). To construct this mutant, the BART miRNA cluster with twenty-two pre-miRNAs was assembled from sub- genomic fragments of the three distinct loci within the BART region (Figure 1A). The loci were PCR-amplified from Jijoye cell DNA and cloned into the expression vector pCDNA3. PCR primers were designed such that DNA stretches of at least 150bp in length flank each of the miRNA loci. Hence, all pre-miRNAs remain in their authentic sequence context minimizing the risk of aberrant RNA folding. This expression cassette was introduced into the *BALF1* gene of prototype 2089 EBV (Figure 1C) as described in detail in the Material and Methods section. *BALF1* encodes a viral homologue of the *Bcl-2* family, and the insertion obliterates its coding capacity but *BALF1* is a redundant gene and therefore dispensable for EBV's transforming functions [31].

Stocks of mutant viruses were generated in HEK293 cells stably transfected with maxi-EBV plasmid DNAs purified from *E. coli* [28]. Virus was produced after lytic cycle induction of the resulting HEK293 producer cell clones and quantified by infecting the B cell Raji cell line as described [32]. Because our recombinant EBVs encode green fluorescence protein (*gfp*), we could measure the concentration of GFP-transducing virions as “green Raji units” (GRU). We obtained virus stocks in the range of 10^4^–10^5^/ml GRUs similar to prototype 2089 EBV stocks [28].

We prepared primary human B cells from three samples of adenoid tissue and two samples of peripheral blood and infected them as described in detail in Material and Methods with prototype 2089 EBV or one of the three miRNA mutant EBVs. We obtained five sets of lymphoblastoid cell lines (LCLs), 20 in total, which we analyzed three to five months post infection (p.i.).

### Steady state levels of selected BHRF-1 and BART miRNAs in established LCLs

**Figure 2:**
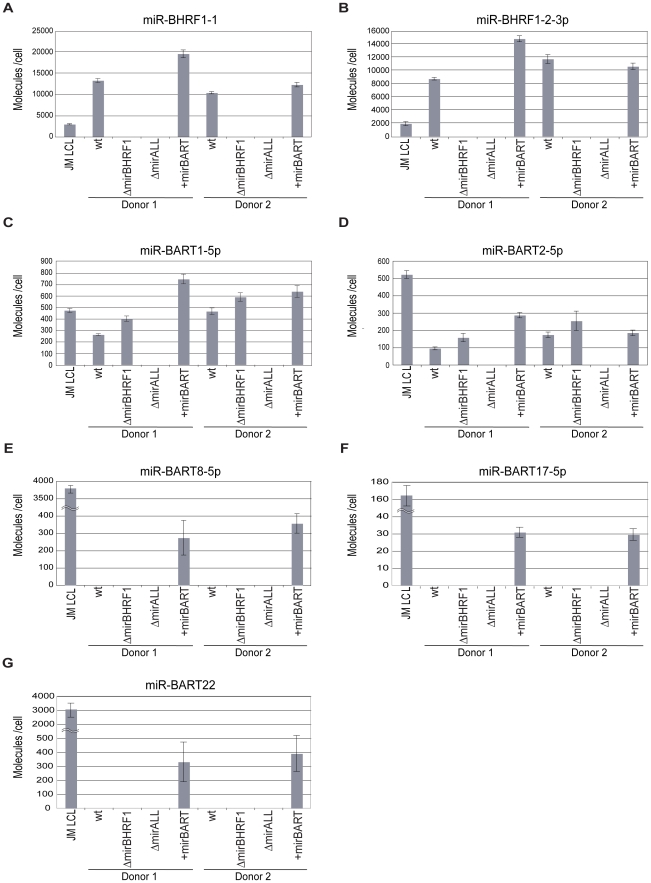
Absolute quantification of EBV-encoded miRNAs in established LCLs infected with prototype 2089 or miRNA-mutant EBVs. Steady-state levels of two BHRF1 miRNAs (panels A, B) and five BART miRNAs (panels C to G) in prototype 2089 or miRNA-mutant EBV-infected LCLs from two different donors were determined by quantitative stem-loop PCR assays. Two BART miRNAs present in the wild-type reference strain B95.8 (panels C, D) and three BART miRNAs deleted in B95.8 but present in +mirBART EBV (panels E to G) were subjected to quantification. JM LCL is a spontaneous LCL infected with an uncharacterized field strain of EBV encoding 44 viral miRNAs. The numbers of miRNA molecules per cell were determined and calculated with standards generated with RNA of ΔmirALL EBV-infected LCLs doped with serial dilutions of synthetic miRNAs. The reconstructions were based on quantification of total RNA harvested from a known number of cells of the different LCLs prior to quantitative stem-loop PCRs. Results are shown as absolute miRNA molecules per RNA mass obtained from a single cell. Data represent the means and standard deviations of three independent quantifications. Prototype 2089 EBV-infected cells are indicated (wt).

We determined the steady state levels of two BHRF1 (Figure 2A, B) and five BART miRNAs (Figure 2C to G) in the established LCLs by quantitative real-time stem-loop PCR analyses. As a positive control, JM LCL was used, an LCL infected with an uncharacterized field strain of EBV that expresses all 44 viral miRNAs. The copy numbers of selected miRNAs per cellular transcriptome were determined with synthetic miRNA standards as references.

Prototype 2089 EBV-infected LCLs expressed BHRF1 miRNAs in the range of 8,000–12,000 copies per cell, which exceeded levels in JM LCL (Figure 2A, B). Expression levels of BHRF1 miRNAs in +mirBART EBV-infected LCLs were in the same range as in prototype 2089 EBV-infected LCL. As expected LCLs infected with ΔmirBHRF1 and ΔmiALL EBVs did not express the functionally deleted miRNAs.

We assessed the expression levels of two BART miRNAs of prototype 2089 EBV (Figure 2C, D) and three BART miRNAs absent in this EBV strain (Figure 2E to G). miR-BART1-5p and miR-BART2-5p were expressed at about 100–500 copies per cell. The relative low expression of BART miRNAs as compared to BHRF1 miRNAs is in accordance with the literature [33 and references therein] and was also observed in JM LCL cells infected with an uncharacterized field strain of EBV. LCLs infected with ΔmirBHRF1 EBV expressed these BART miRNAs at levels similar to prototype 2089 EBV-infected LCLs. +mirBART EBV infection mildly increased the levels of miR-BART1-5p and miR-BART2-5p (Figure 2C, D). Steady state levels of those miRNAs absent in B95.8-derived EBVs were considerably lower in +mirBART EBV-infected cells than in JM LCL cells (Figure 2E to G).

### EBV's miRNAs have no discernable role in maintaining viral latency

**Figure 3:**
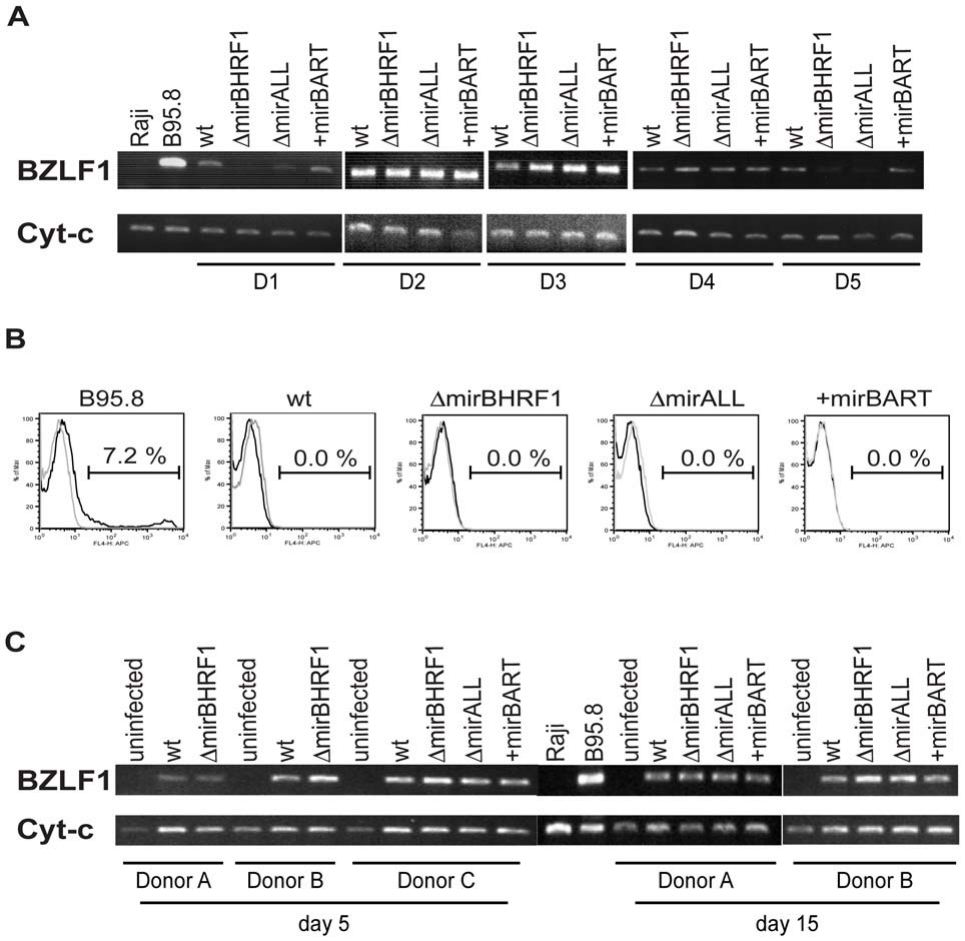
Expression of *BZLF1* and *BLLF1* in prototype 2089 or miRNA mutant EBV-infected LCLs. (A) Semi-quantitative RT-PCRs of transcripts of the immediate-early gene *BZLF1* in established LCLs from five different donors (D1 to D5) infected with prototype 2089 EBV (wt) or the indicated miRNA mutants. cDNAs prepared from B95.8 and Raji cells served as positive and negative controls, respectively. The levels of cytochrome c mRNA were used as loading controls. (B) FACS analysis of the surface expression of gp350/220 encoded by the late EBV gene *BLLF1*. LCLs were stained with a mouse anti-gp350 monoclonal antibody, followed by APC-conjugated goat anti-mouse IgG antibody (black histograms). The gray histograms represent the staining of the secondary antibody, only. A 7.2% fraction of B95.8 cells spontaneously supports EBV's lytic phase. One representative set of LCLs from one out of five different donors is shown. (C) Semi-quantitative RT-PCR analysis of BZLF1 transcripts in primary B cells at day 5 and 15 p.i. infected with prototype 2089 EBV (wt) or the indicated miRNA mutant EBVs. Primary human B cells isolated from adenoids were infected at a concentration of 4.5×10^5^ per ml with an MOI of 0.2.

Viral miRNAs have been implicated in maintaining herpesviral latency by inhibiting induction of the lytic cycle [34 for a recent review]. We therefore asked whether deleting EBV's miRNAs might lead to spontaneous induction of EBV's lytic phase in LCLs. We analyzed the expression of *BZLF1* by semi-quantitative RT-PCR and the expression of *BLLF1* by FACS in established LCLs. *BZLF1* is the molecular switch gene of EBV, which can induce EBV's lytic phase, and *BLLF1* codes for the late structural glycoprotein gp350/220 expressed on the surface of productively infected cells. The expression levels of *BZLF1* transcripts did not consistently differ between B cells infected with prototype 2089 or miRNA mutant EBVs (Figure 3A). *BLLF1* as a late lytic gene was also not detectably expressed in established LCLs infected with miRNA mutant EBVs (Figure 3B) indicating that EBV's miRNAs are not essential for maintaining herpesviral latency or inhibiting spontaneous reactivation of EBV's lytic phase in established cell lines.

We were surprised to learn that obliterating EBV's miRNAs did not lead to increased lytic reactivation given that other herpesvirus such as KSHV, HSV, and CMV have been reported to encode miRNAs, which maintain and stabilize latent infection [35]–[39]. We therefore analyzed the expression of BZLF1 mRNAs by semiquanitative RT-PCR in primary B cells from three different donors infected with the different mutant EBVs five and 15 days p.i.. Again, no discernable differences were seen indicating that BZLF1 transcripts are not regulated by EBV's miRNAs (Figure 3C). At early time points post infection *BZLF1* is expressed at relative high levels [40], [41] but nevertheless cannot induce EBV's lytic phase [41]. Our recent findings together suggest that in contrast to other members of the herpesvirus family [34] EBV does not rely on its miRNAs but uses alternative means to control establishment or maintenance of latency.

### Functional deletion of BHRF1 miRNAs causes minor alterations in cell cycle distribution but does not affect survival of established LCLs

While cultivating the twenty LCL lines for up to five months p.i. we noticed that LCLs infected with ΔmirBHRF1 and ΔmirALL proliferated slightly slower than LCLs infected with prototype 2089 or +mirBART EBVs (data not shown). We therefore analyzed the phenotypes of viral miRNA EBV mutants in established LCLs by monitoring their cell cycle distribution and the fraction of apoptotic cells.

**Figure 4:**
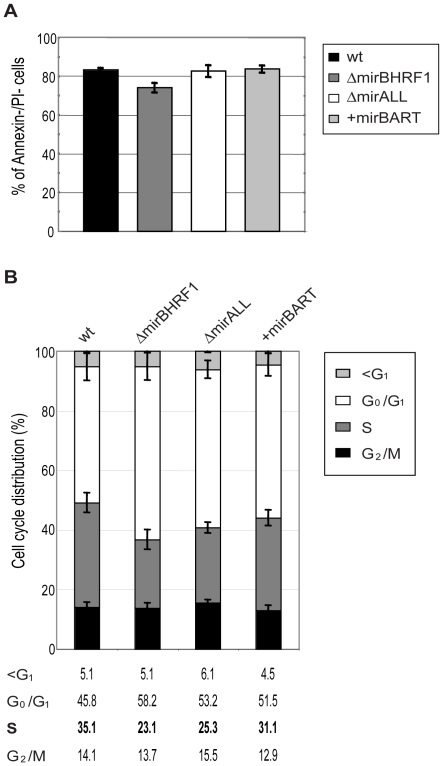
Established LCLs infected with BHRF1 miRNA mutant EBVs show an altered cell cycle distribution but no survival phenotype. Long-term (three to five months p.i.) cultured cells infected with either prototype 2089 or miRNA-mutated EBVs were analyzed for spontaneous apoptosis and cell cycle distribution. (A) Established LCLs were plated (10^5^ per ml) in fresh medium and cultured for two days. The cells were analyzed by FACS for Annexin-V and PI staining. The error bars represent the standard deviation of the mean of cells in this experiment from five different donors. (B) Cell cycle analysis of established LCLs. Cells were plated (10^5^ per ml) in fresh medium and cultured for two days, then analyzed by standard BrdU incorporation assays. The error bars represent the standard deviation of the mean of cells in this experiment from four different donors.

The 20 LCL lines were plated at initial cell densities of 10^5^ cells per ml and cultured for 2 days. Surface staining for Annexin-V, uptake of propidium iodine (PI), and FACS analyses of their DNA content after BrdU incorporation revealed the fraction of living cells and their cell cycle distributions, respectively. The proportions of cells that were double negative for Annexin-V and PI staining ranged between 75 to 85% for LCLs infected with either prototype 2089 or miRNA mutant EBVs (Figure 4A) with no discernable differences. LCLs infected with ΔmiBHRF1 and ΔmirALL mutant EBVs showed a slightly increased proportion of cells in G_0_/G_1_ with a reduction of cells in S phase when compared to prototype 2089 EBV-infected LCLs (Figure 4B). This tendency was mostly statistically significant (Supporting Figure S2) and suggested a possible role of EBV's BHRF1 miRNAs in controlling proliferation in latently infected cells.

A previous, detailed genetic analysis of the *BHRF1* gene, a viral homologue of the cellular Bcl-2 family, had not revealed a measurable phenotype in latently infected primary human B cells [31]. *BHRF1* is a redundant gene because EBV carries two alleles of Bcl-2 family members, *BHRF1* and *BALF1*, which are both highly expressed shortly after infection. Singly inactivated *BALF1^−^* or *BHRF1^−^* mutant EBVs were capable of yielding clonal LCLs but at a slightly higher dose than wild-type EBV. Only viral mutants with two inactivated *Bcl-2* genes (i.e. both *BALF1* and *BHRF1*) failed to rescue infected primary B lymphocytes from spontaneous apoptosis, prevented their cell cycle entry and did not generate LCLs [31]. Thus, either *BHRF1* or *BALF1* is dispensable for growth transformation by EBV because the two viral vBcl-2 members encode similar functions.

A recent publication indicated that *BHRF1* is constitutively expressed as a latent protein in growth-transformed cells *in vitro* and may contribute to virus-associated lymphomagenesis *in vivo* [42]. Therefore, we were concerned that our current findings might result from reduced steady-state transcript levels of BHRF1 mRNA, which could be the consequence of the altered pre-miRNA sequences located in the 5′ and 3′ untranslated regions of that mRNA (Figure 1A). The alterations could adversely affect mRNA translation and reduce BHRF1 protein levels. Because antibodies that unambiguously detect BHRF1 protein early after infection or in strictly latently infected LCLs are not available, we assessed BHRF1 mRNA levels by quantitative RT-PCR analyses in established LCLs or primary B cells infected with prototype 2089 EBV or ΔmirBHRF1 mutant EBVs for five days, only (Supporting Figure S3). No discernable differences in LCLs were observed (Supporting Figure S3A and data not shown) but primary B cells infected with ΔmirBHRF1 revealed slightly enhanced (up to twofold) levels of BHRF1 mRNA early after infection (Supporting Figure S3B and data not shown). Our attempts to directly detect BHRF1 protein in newly infected primary B cells failed (data not shown). Low protein expression levels or the insufficient sensitivity of available antibodies prevent the detection of BHRF1 at early time points after infection but our quantitative RT-PCR results indicated that scrambling of the untranslated BHRF1 pre-miRNA sequences upstream and downstream of the BHRF1 coding sequence did not negatively affect the expression levels of this transcript. On the contrary the genetic alterations might even improve the expression or stability of the BHRF1 transcripts up to twofold (Supporting Figure S3B), which is expected to result in mildly enhanced protein levels that should counteract apoptosis in latently and newly infected primary B cells [31], [42 and references therein].

### Functional deletion of BHRF1 miRNAs reduces early B-cell proliferation

Our studies with established LCLs infected with BHRF1 miRNA mutant EBVs indicated that these cells differed only in their cell cycle distribution but lacked other obvious phenotypes. We have found that the very early but transient expression of several lytic viral genes in primary human B cells is critical for their subsequent transformation and stable latent infection [31], [41]. *BHRF1* is among the genes that are massively expressed initially after infection [31], [42]. It codes not only for one of the two viral *Bcl-2* homologous but also for three pre-miRNAs that give rise to the four mature miRNAs of the *BHRF1* locus (Figure 1A) [33 and references therein]. We suspected that the strong, initial expression of this gene might have implications for the expression and function of the encoded miRNAs and therefore examined their expression and the proliferation of primary B cells infected with prototype 2089 EBV or miRNA mutant EBVs at early time points post infection.

First, primary B cells prepared from adenoids were infected with prototype 2089 EBVs with a high multiplicity of infection (MOI) of 0.2 to ensure infection of many primary B cells. Quantitative stem-loop PCR analyses assessed the absolute levels of two miRNAs, miR-BHRF1-1 and miR-BHRF1-2-3p at day 5 p.i.. In primary cells miR-BHRF1-1 and miR-BHRF1-2-3p were expressed at about four- and twofold higher levels, respectively, early after infection (Supporting Figure S4; panels A and B) as compared to established LCLs infected with the same prototype 2089 EBV (Figure 2A, B). The levels early after infection exceeded the steady state levels of BHRF1 miRNAs seen in the reference JM LCL infected with an uncharacterized field strain of EBV (Supporting Figure S4; panels A and B). Similar findings apply to the expression levels of BART miRNAs early after infection (Supporting Figure S4), which were in a similar range or even exceeded those seen in JM LCL cells (miR-BART2-5p, miR-BART22, miR-BART1-5p; Supporting Figure S4C, D, F) with one exception (miR-BART8-5p; Supporting Figure S4E).

**Figure 5:**
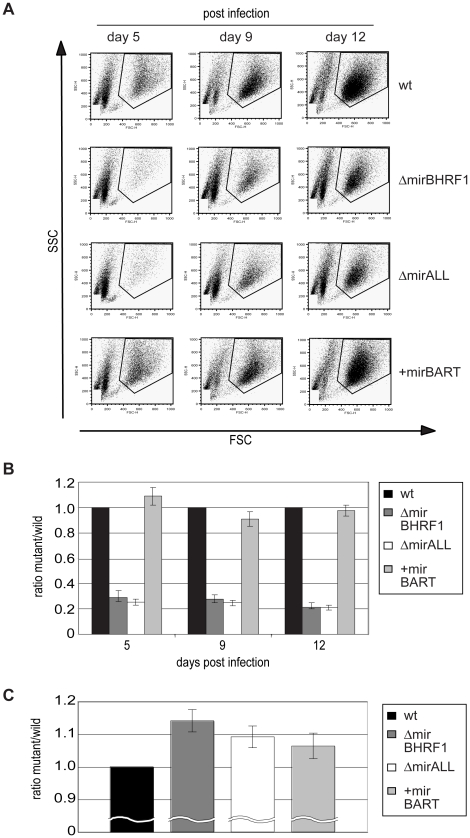
Outgrowth of primary human B cells infected with prototype 2089 or miRNA-mutated EBVs. Primary human B cells of three donors isolated from adenoids (4.5×10^5^ per ml) were infected with either prototype 2089 or miRNA-mutated EBVs at an MOI of 0.05. At day 5, 9, and 12 p.i., cells were harvested and their outgrowth analyzed by FACS. To determine the absolute number of cells counted, a volume standard was added prior to FACS analysis as described [31]. (A) Representative results of FACS analysis. EBV-infected cells with elevated forward (FSC) and sideward (SSC) scatter characteristics indicative of activated lymphoblast cells were gated as indicated and the number of cells in this gate was recorded. (B) The ratios of the numbers of lymphoblastic cells infected with single miRNA mutant EBVs versus prototype 2089 EBV-infected cells were calculated. Functional deletion of EBV-encoded BHRF1 miRNAs led to substantially lower numbers of outgrowing lymphoblasts compared to wild-type EBV-infected cells. The error bars represent the standard deviation of the mean of cells from three different donors. (C) Doubling times of primary B cells infected with either prototype 2089 or miRNA-mutated EBVs calculated from the results in (B).

Next, primary B cells prepared from adenoids were infected with viral stocks having identical titers of the three different mutant EBVs, ΔmirBHRF1, ΔmirALL, and +mirBART EBV and prototype 2089 EBV with an multiplicity of infection (MOI) of 0.05 and a concentration of 4.5×10^5^ cells per ml for 18hours. After collection and resuspension in fresh medium to the initial density, the infected cells were cultivated and analyzed by FACS at 5, 9, and 12 days p.i. (Figure 5A). Uninfected primary B cells showed the typical forward (FSC) and sideward (SSC) scatter characteristic of small and resting cells. Infected cells acquired typical lymphoblastic characteristics of activated cells, increased their forward and sideward scatter and were found in the defined LCL gate (Figure 5A, top panels). The absolute numbers of cells in this gate were determined by FACS counting with the aid of added APC-coupled calibration beads as a volume standard as described [31]. After infection with the two miR-BHRF1-negative EBVs, ΔmirBHRF1 and ΔmirALL, fewer cells were present in the LCL gate than after infection with prototype 2089 EBV as early as 5 days p.i. In contrast, the number of cells infected with +mirBART EBV was in a similar range as prototype 2089 EBV (Figure 5A). The inactivation of the BHRF1 miRNAs led to a four to five-fold reduction in outgrowth of B cells from three different donors (Figure 5B). The reduced numbers of growing cells infected with ΔmirBHRF1 or ΔmirALL EBVs were consistently observed over 12 days p.i.. Cells infected with ΔmirBHRF1 and ΔmirALL EBVs showed a slightly prolonged doubling time, which was not significantly different (p≥0.1, paired t test; data not shown) when compared to prototype 2089 or +mirBART EBV-infected cells (about 2 to 2.5 days/cell generation; Figure 5C). These combined observations showed that BHRF1 miRNAs are critical in primary B cells early after infection but largely dispensable in established LCLs.

### BHRF1 miRNAs protect primary B cells from spontaneous apoptosis early after infection

**Figure 6:**
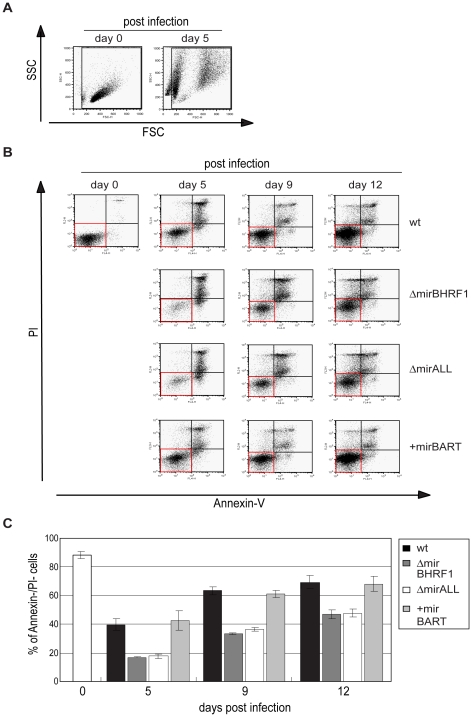
Spontaneous apoptosis of primary B cells infected with prototype or miRNA-mutated EBVs. To examine the spontaneous apoptosis of primary B cells early after infection, the samples as shown in Figure 5 were analyzed by FACS for Annexin-V binding and propidium iodide (PI) staining at different time points p.i.. (A) FSC and SSC characteristics of uninfected and prototype 2089 EBV-infected primary B cells on day 0 and day 5 p.i., respectively. To exclude subcellular debris but include highly granular, presumably dying or dead cells, only cells within the indicated gate were analyzed for their Annexin-V and PI staining. (B) Annexin V/PI staining analysis of B cells infected with 2089 EBV or miRNA mutants at different time points p.i.. One representative experiment out of three is shown. Cells in the AnnexinV^−^/PI^−^ quadrants (red boxes) indicate surviving cells at each time point p.i.. (C) Summary of the data shown in (B). The proportion of live, AnnexinV^−^/PI^−^ cells at each time point p.i. was calculated. Fewer live cells were detected after ΔmirBHRF1 or ΔmirALL EBV infection than after prototype 2089 or +mirBART EBV infection indicating an anti-apoptotic role of the BHRF1 miRNAs during the early phase of B cell infection. The error bars represent the standard deviation of the mean of three different experiments.

Our initial findings indicated that BHRF1 miRNAs support B cells early after EBV infection but did not distinguish between BHRF1 miRNAs′ regulating cell cycle functions or counteracting the spontaneous apoptosis of primary B cells. To differentiate between these two roles, we first determined the proportion of viable cells in miRNA mutant or prototype 2089 EBV-infected B cells at different time points early after infection. Forward and sideward scatter analysis of primary B cells immediately after preparation showed mostly intact cells with a minor fraction of subcellular debris (Figure 6A, left panel). Uninfected primary B cells die rapidly *in vitro* [31], whereas EBV-infected cells become lymphoblastoid with characteristically increased forward and sideward scatter (Figure 6A, right panel). We deliberately chose a low MOI (0.05) in order to detect small changes in the fraction of infected and surviving B cells. As a consequence the majority of cells were uninfected, became highly granular or disintegrated in the course of infection. The gate in Figure 6 was set in order to include primary and activated, live and apoptotic cells but to exclude most of the subcellular debris. The cells in this gate were analyzed for the binding of Annexin-V and uptake of PI as indicators of early apoptosis and loss of membrane integrity, respectively.

Measuring the percentage of cells, which were double-negative for Annexin-V and PI staining indicated that nearly 90% of the cells were alive on day 0 (Figure 6C). Infection with prototype 2089 EBV yielded up to 70% of cells, which were Annexin-V- and PI-negative twelve days p.i.. Both EBV mutants, ΔmirBHRF1 and ΔmirALL, also rescued the infected cells from cell death but considerably less efficiently than prototype 2089 EBV (Figure 6C). As already pointed out, we cannot unambiguously dissect BHRF1 protein-mediated effects from BHRF1 miRNA-mediated effects due to a lack of antibody reagents. However, the genetic and functional redundancy of EBV's anti-apoptotic Bcl-2 homologs [31] and the analysis of the levels of the BHRF1 transcript (Supporting Figure S3) clearly point to miRNAs of the *BHRF1* cluster and their role in inhibiting apoptosis.

The larger fraction of apoptotic cells infected with the two mutant EBVs likely contributes to some of the reduction in numbers of proliferating cells in Figure 5 consistent with BHRF1 miRNAs contributing to cellular proliferation by supporting initial B-cell survival. We did not observe a clear difference between prototype 2089 EBV- and +mirBART EBV-infected cells, as would be expected from their similar growth characteristics (Figure 5).

### BHRF1 miRNAs affect cell cycle distribution of infected B cells

**Figure 7:**
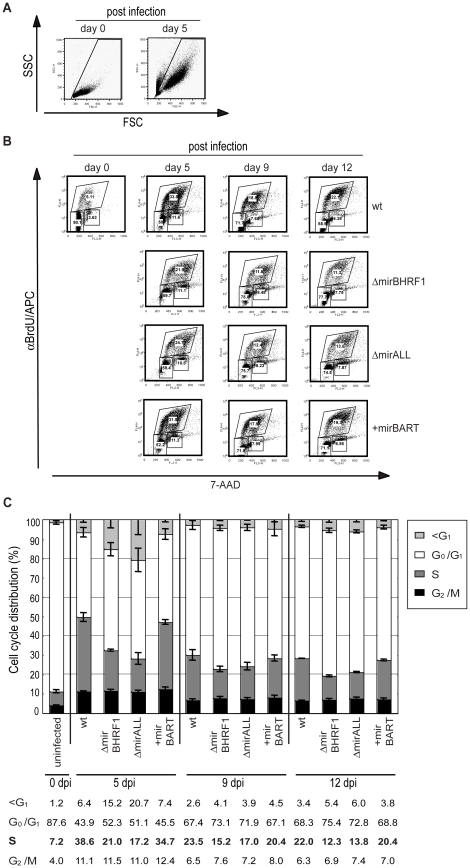
Cell cycle distribution of primary human B cells infected with miRNA-mutant or prototype 2089 EBVs. Primary B cells (4.5×10^5^ per ml) were infected with an MOI of 0.05 of each virus stock as indicated or left uninfected. BrdU incorporation, 7-AAD counterstaining and FACS analysis determined the cell cycle profiles of cells with characteristics of resting lymphocytes and activated lymphoblasts. (A) FSC and SSC distribution characteristics of uninfected and prototype 2089 EBV-infected primary B cells on day 0 and day 5 p.i.. To exclude subcellular debris and highly granular, presumably dying or dead cells, only cells within the indicated gate were analyzed. (B) FACS analysis for their BrdU and 7-AAD staining. One representative experiment out of three is shown. Gates used to define cells in G_1_/G_0_, S and G_2_/M are indicated. (C) The compiled data (mean values and standard deviations) of three experiments as exemplified in panel B indicate the different cell cycle profiles of infected B cells at three time points p.i.. Cells infected with ΔmirBHRF1 or ΔmirALL EBV mutants showed an increased fraction of cells in G_0_/G_1_ and a concomitant decrease of cells in S as compared to cells infected with prototype 2089 EBV or +mirBART mutant EBV. Likewise, more cells were found in the subG_1_ fraction when the cultures were infected with ΔmirBHRF1 or ΔmirALL EBV mutants. The differences in cell cycle distributions were most obvious early after infection on day 5 and diminished thereafter.

Cells infected with ΔmirBHRF1 and ΔmirALL EBVs showed slightly prolonged doubling times (Figure 5C). We employed BrdU incorporation assays to compare the cell cycle distributions of the differently infected B cells early after infection as accurately as practical. The cell cycle status of uninfected (day 0) or infected cells on day 5, 9, and 12 p.i. with characteristics of resting lymphocytes and activated lymphoblasts in forward/sideward scatter analysis (Figure 7A) was analyzed by determining BrdU incorporation and 7-AAD uptake by FACS (Figure 7B). On average 7.2% of the uninfected cells were in S-phase immediately after isolation (Figure 7C). Whereas uninfected cells died rapidly, 39% or 35% of prototype 2089 or +mirBART EBV-infected cells were in S phase five days p.i.. In contrast, ΔmirBHRF1 and ΔmirALL EBV-infected cell cultures contained fewer cells in S-phase (21% or 17%, respectively) consistent with an increase in cells in G_0_/G_1_ and a higher proportion of apoptotic cells (Figure 7C). Thus, it appears that BHRF1 miRNAs both promote cell cycle entry or progression and block apoptosis in B cells early after their infection.

## Discussion

EBV is now thought to encode more miRNAs than do other herpesviruses yet little has been established about the roles of these regulatory genes in EBV's life cycle. We have used genetic analysis to identify phenotypes mediated by the *BHRF1* cluster of miRNAs. Derivatives of EBV lacking these miRNA genes yielded established B cell lines that behaved as did those infected with wild-type virus. However, careful scruting of B cells immediately following infection has uncovered two complementary functions that the BHRF1 miRNAs provided these cells. By five days post infection twice the numbers of cells infected with EBV lacking the BHRF1 miRNAs were undergoing apoptotic death as were those infected with EBV encoding these miRNAs. At this same time twice as many viable cells infected with EBV encoding the BHRF1 miRNAs were in S phase as were those infected with EBV lacking these miRNA genes. These findings indicate that the BHRF1 miRNAs inhibit apoptosis and promote proliferation during the early stages of infection. They are thus acting at a stage in the life cycle when EBV's multiple oncogenes are only beginning to function. The EBV-mediated differentiation of the resting B cell to the proliferating B cell blast requires multiple days following infection at low multiplicity, a scenario likely to reflect infections *in vivo*. It is under these circumstances that the BHRF1 miRNAs contribute substantially to promoting survival and proliferation of the infected B cell.

The functions of EBV's BHRF1 miRNAs differ from those characterized in KSHV's genome. In KSHV, an EBV-related human herpesvirus, several miRNAs counteract the spontaneous onset of KSHV's lytic cycle. Their expression promotes or maintains viral latency and shuts off viral lytic proteins. For example, two groups recently demonstrated that two different miRNAs of KSHV, miRK9* and miRK5, can down-regulate the expression of the viral transcription activator RTA [27], [35]. Another miRNA of KSHV, miR-K1, negatively regulates the IκBα protein level to increase NF-κB activity and indirectly inhibit viral lytic replication in certain cells [36]. It is tempting to speculate that the role of KSHV's miRNAs is not only to maintain latency but also to prevent the spontaneous expression of viral lytic genes, which, by analogy with EBV, might otherwise increase the susceptibility of virus-infected cells to T effector cells. Similar results have been obtained while studying the functions of miRNAs encoded in CMV and HSV, which also help maintain latent infection [38], [39].

A subset of BART miRNAs can negatively regulate the viral oncoprotein LMP1 (latent membrane protein 1) in nasopharyngeal carcinoma cells [43]. LMP1 has transforming activity but its high expression can cause growth inhibition and apoptosis. LMP1 regulates its level through its regulation of the Unfolded Protein Response (UPR) pathway and autophagy. EBV's miRNAs might limit inappropriately high LMP1 levels and thereby prevent apoptosis resulting from LMP1's regulation of the UPR [44]–[46]. It has also been shown that the pro-apoptotic protein PUMA (p53-upregulated modulator of apoptosis) is a target of miR-BART5 when expressed in epithelial cells, which prototype 2089 EBV does not encode (Figure 1). We investigated the expression levels of LMP1 and PUMA in 20 LCLs infected with prototype 2089 or miRNA mutant EBVs but did not observe a consistent correlation with the expression levels of LMP1 protein or PUMA mRNA (data not shown). The relative low expression levels of the BART miRNAs in the reconstituted EBV mutant +mirBART in established LCLs might not be sufficient to reveal phenotypes that correlate with the regulation of the two proteins in B cells with a latency III type program (Figure 2C–G). The low steady state levels might also mask other phenotypes that might be connected to viral reactivation or additional phenotypic effects not diclosed in this work. This caveat is of concern because the BART miRNAs are expressed at considerably higher levels in nasopharyngeal carcinoma cells [47]. Early after infection, these BART miRNAs (as well as BHRF1 miRNAs) are expressed at much higher levels (Supporting Figure S4). In fact, we observed under conditions of low cell density and reduced multiplicity of infection that the ectopically expressed BART miRNAs of EBV do promote proliferation of primary human B cells early after infection (Vereide et al., manuscript submitted).

It is likely that many targets of viral miRNAs remain to be identified because single miRNAs can target multiple mRNAs [11]. Computer algorithms based on the conservation of seed sequences between different species have been successfully used for the target prediction of cellular miRNAs, but this approach is hampered for the prediction of the targets of herpesviral miRNAs because they are evolutionarily poorly conserved [14] and do not share extended seed homology with cellular transcripts [9 and references therein]. Conversely, multiple miRNAs could simultaneously downregulate a single target gene even if the individual miRNAs are expressed at relatively low levels [48]. Therefore, the common experimental approach based on the ectopic expression or repression of individual miRNAs coupled to subsequent microarray analysis may prove inadequate to identify targets for herpesviral miRNAs. Given these difficulties it is essential to identify phenotypes mediated by EBV's miRNAs when they are expressed at physiological levels under normal conditions of infection as we have done. It is particularly intriguing that these genetic analyses show that EBV has evolved miRNAs to support its defining phenotype of transforming infected B cells.

## Materials and Methods

### Construction of miRNA mutant EBVs

EBVs used in this study were derived from p2089, which comprises the B95.8 EBV genome cloned onto an F-factor plasmid in *E. coli* [28]. The B95.8 EBV strain as well as p2089 encode a total of 13 known miRNAs, which are located in four clusters (Figure 1; miR-BHRF1-1, miR-BHRF1-2/3, miR-BART3/4/1/15/5, and miR-BART2). For their functional ablations, the wild-type miRNA sequences in each of the four clusters were replaced with computed, scrambled miRNA sequence (Table 1) in four consecutive rounds of homologous recombination with the *galK*-based recombineering system [30]. In a two-step approach this system allows modifying the p2089 genome via homologous recombinations in *E. coli* without permanently introducing selectable marker genes or cis-acting sequences (or their remnants) at the sites of genetic alterations.

Briefly, the recombineering *E. coli* strain SW105 has a deletion of the galactokinase (*galK*) gene and carries a lysogenic and temperature-sensitive λ prophage that makes recombination amenable. We introduced the p2089 plasmid into SW105 by electroporation.

In the first targeting step, we wanted to replace the miR-BHRF1-2/3 miRNAs in p2089 with their scrambled counterparts shown in Table 1. In the first step we inserted the *galK* gene into the miR-BHRF1-2/3 cluster deleting the entire locus. To achieve this step, the *galK* targeting cassette was PCR amplified with the p*galK* plasmid as a template [30] with the following conditions: 94°C for 3min for initial denaturation, 94°C for 45sec, 54°C for 45sec, and 72°C for 2.5min in 15 cycles, followed by 20 cycles at 94°C for 45sec, 62°C for 45sec, and 72°C for 2.5min, and a final elongation step at 72°C for 3min. The PCR primers suitable for replacing each of the miRNA cluster with *galK* are listed in Supporting Table S1. After DpnI digestion, the gel-purified PCR fragment was electroporated into the heat-induced and therefore recombination-competent *E. coli* SW105 strain carrying the plasmid p2089. After selection for *galK* on minimal medium plates with galactose as the sole carbon source, plasmid DNAs were prepared from *galK* positive bacterial clones and carefully analyzed by restriction enzyme analysis. The resulting EBV plasmid p3994 was confirmed to carry *galK* replacing the BHRF1-2/3 miRNA cluster.

The second targeting step aimed at replacing *galK* with designed scrambled DNA sequences that maintain the original nucleotide composition but ablate the original pre-miRNA structures. The targeting constructs consisted of the scrambled pre-miRNA sequence as the core flanked by 150–200 bp long homologous arms on both sides for the efficient and precise replacement of *galK*. The targeting constructs were custom-made, synthetic DNA fragments cloned into pUC57 and obtained from a commercial service provider (Genscript Corporation). Four targeting constructs were ordered. To replace the *galK* gene inserted into the mir-BHRF1-2/3 locus the targeting p3969 plasmid was cut with appropriate restriction enzymes to liberate the synthetic DNA fragment. It was gel-purified and electroporated into recombination-competent *E. coli* SW105 cells carrying p3994, which were selected for loss of *galK* by growth on minimal plates containing 2-deoxy-galactose (DOG) and glycerol as carbon sources as described in detail [30]. Plasmid DNAs were prepared from *galK* negative bacterial clones and carefully analyzed by restriction enzyme analysis and extensive DNA sequencing covering at least two kbps of upstream and downstream flanking sequences in order to verify the correct insertion of the scrambled miRNA sequences.

We repeated the two-step approach and replaced the miR-BHRF1-1 cluster with scrambled sequences to generate the genomic EBV plasmid p4004, which lacks the four miRNAs of the BHRF1 locus. With this genomic EBV plasmid ΔmirBHRF1 EBV stocks were established and calibrated as described in detail [32].

On the basis of p4004, the two BART miRNA clusters in the cloned genome of B95.8 EBV were further replaced with the scrambled sequences shown in Table 1. The resulting genomic EBV plasmid p4027 lacks all functional viral miRNAs (ΔmirALL EBV). Restriction enzyme analysis and partial DNA sequencing as exemplified above verified the genetic compositions of the modified EBV genomes.

The B95.8 EBV strain and the derived genomic EBV plasmid p2089 encompass only 13 out of 44 miRNAs as compared to EBV field strains, which encode 31 additional BART miRNAs. To reconstitute an EBV genome that has the miRNA coding capacity of EBV field strains, we introduced an expression cassette, termed pCMV-miRBART, encompassing all known 22 BART pre-miRNAs driven by human CMV promoter into the *BALF1* locus of p2089 by homologous recombination (Figure 1C). An expression cassette was assembled from three sub-genomic fragments containing the BART miRNAs that map to three distinct loci within the BART region (Figure 1A). These loci were PCR amplified from Jijoye cellular DNA and inserted into the expression vector pCDNA3 (Invitrogen), followed by sequencing to ensure accurate DNA amplification. Primers were designed such that DNA stretches of at least 150bp in length flank each of the miRNA loci. The expression cassette was termed pCMV-miRBART. The nucleotide positions of the amplified regions were as follows (all positions are given in reference to GenBank entry AJ507799): nucleotide coordinates #138803 to #140353 (containing miRs BART1, −3 to −6, −15 to −17), nucleotide coordinates #145331 to #149070 (miRs BART7 to −14, −12 to −14, −18 to −22) and nucleotide coordinates #152509 to #153034 (miR-BART2). In order to ensure proper function of the construct, we verified the expression of representative miRNAs from each of the inserted loci in transiently pCMV-miRBART-transfected cells by northern blot hybridization confirming similar relative expression levels as in wild-type EBV infected B-cell lines (data not shown).

To construct the maxi-EBV genome p4080 (+mirBART in Figure 1) a tetracycline resistance gene was introduced into the NruI site of pCMV-miRBART (p3971) to yield p4016. The final targeting plasmid p4079 was generated by inserting the SspI/DrdI fragment from p4016 cloned into the SmaI site of p2642, which contains the *BALF1* gene to support its targeted homologous integration into the *BALF1* locus as shown in Figure 1C. The targeting construct p4079 was linearized with BsrDI/BssHII digestion and electroporated into the SW105 strain carrying p2089. After tetracycline selection and restriction enzyme analysis, DNA sequencing confirmed the genomic EBV plasmid p4080 to contain the entire targeting construct at the desired location in the *BALF1* locus.

### Cells and culture

The EBV-positive Burkitt's lymphoma cell line Raji, the EBV-positive marmoset cell line B95.8, and HEK293 cells were maintained in RPMI 1640 medium (GIBCO). All media were supplemented with 10% FBS (PAA laboratories), penicillin (100 U/ml), and streptomycin (100µg/ml). Cells were cultivated at 37°C in a 5% CO_2_ incubator.

### Preparation and quantification of infectious viral stocks

On the basis of HEK293 cells, virus producer cell lines were established after individual transfection of the genomic EBV plasmid DNAs and subsequent selection with hygromycin (80µg/ml). To obtain virus stocks, the producer cell lines were transiently transfected with expression plasmids encoding *BZLF1* [49], *BALF4* [50], and *BRLF1* [51] to induce EBV's lytic cycle. Three days post transfection, supernatants were harvested and centrifuged at 3000rpm for 15min to remove cell debris. The titers of the different virus stocks were quantified and the concentrations of GFP-transducing virions expressed as “green Raji units” (GRUs) were determined as described previously [31]. Briefly, 10^5^ Raji cells were incubated with serial dilutions of virus stocks at 37°C for 24hours. After an additional culture for 2 days, the percentage of GFP positive cells was determined by FACS using a FACS-Calibur instrument (Becton Dickinson).

### Isolation, separation, and infection of human primary B lymphocytes

Human primary B cells from adenoids were separated from T cells by rosetting with sheep erythrocytes and purified by Ficoll-Hypaque density gradient centrifugation. B cells isolated from human peripheral blood mononuclear cells (PBMC) by Ficoll-Hypaque gradient centrifugation were purified using the B-cell isolation kit II (Miltenyi Biotec) and MACS separators (Miltenyi Biotec). For virus infection, primary B cells were incubated with each virus stock for 18 hrs. After replacement with fresh medium, the infected cells were seeded at an initial density of 4.5×10^5^ cells per ml.

Anonymized adenoid tissue samples from routine adenoidectomies were provided by the Department of Otorhinolaryngology, Klinikum Grosshadern, Ludwig-Maximilians-University of Munich. The institutional review board, Ethikkommission of the Klinikum Grosshadern, approved of the study and did not require prior informed patient consent.

### Analysis of cell proliferation, spontaneous apoptosis and cell cycle distribution of infected primary B cells

For each time point p.i., 1ml of each cell sample was collected by centrifugation and stained with 2µl of AnnexinV-Cy5 reagent (BioVision) and 50µg of propidium iodide (PI) in 250µl of Annexin-V binding buffer (BioVision) for 5 min at room temperature. As an internal FACS volume standard, 2×10^4^ of APC-conjugated BD CaliBRITE beads (Becton-Dickinson) were added. The beads are small, resulting in a high intensity in the sideward scatter channel, and do not interfere with the cells to be analyzed. Cells were analyzed by FACS until 5,000 beads were counted and the numbers of the cells in the indicated gates were recorded.

For analysis of the cell cycle status, the infected cells were incubated with the thymidine analog BrdU for 2hrs prior to FACS analysis at each time point. The cells were stained with an APC-coupled BrdU-specific antibody after fixation and permeabilization, and the cellular DNA was counter-stained with the DNA intercalating dye 7-AAD according to the manufacturer's protocol (BrdU Flow Kit, BD Biosciences Pharmingen). 3×10^4^ cells were acquired in FACS analysis. The FACS data were gated for cells in the G_0_/G_1_, S, G_2_/M phases of the cell cycle and for cells with a subG_1_ DNA content. The total of all events was set to 100%.

### Absolute quantification of EBV-encoded microRNAs by stem-loop real-time PCR

Total RNA was extracted from infected cells using the Trifast reagent (peqGOLD). For cDNA synthesis, 500 ng of total RNA was reverse-transcribed using Superscript III reverse transcriptase (Invitrogen) with the mixture of miRNA specific stem-loop primers shown in Supporting Table S2. In the quantitative reconstruction experiments, total RNA from ΔmirALL EBV-infected LCLs was used as an EBV miRNA-negative but complex RNA sample in reverse transcription (RT) reactions. Details for reverse transcription reaction were described previously [52]. 4ng of cDNA aliquots from each sample were subjected to quantitative real-time PCR analysis. Each 10µl PCR reaction contained 0.5µM forward primer, 0.5µM reverse primer, and 1×SYBR green mix (Roche). The PCR reaction was performed in 96-well cluster plates at 95°C for 10min, followed by 45 cycles of 95°C for 15sec, 60°C for 1min with 10 initial cycles of touchdown steps (70–60°C). The absolute copy number of each miRNA in the test samples was reconstructed with the aid of standard curves generated with the serial dilution of synthetic miRNA (Metabion) as reference. The synthetic miRNAs were identical to the mature miRNA sequences as annotated in the microRNA database (miRBase, release 14, Sept. 2009; http://www.mirbase.org).

## Supporting Information

Figure S1 Alignments and predicted structures of mutant miRNAs. This multi-page figure shows alignments (pages 1 to 6) and predicted secondary structure images (pages 7 to 13) of the 25 pre-miRNAs encoded by EBV field strains (represented by the EBV GenBank entry AJ507799) and the corresponding sequences in the mutant strains (ΔmirBHRF1, ΔmirALL, +mirBART) as well as the prototypic parental 2089 EBV strain (wt), which is a molecular clone of the EBV B95.8 genome. Secondary structures were predicted using the Vienna RNAfold package [53] and are indicated by bracket notation above and below the aligned sequences. Regions that encode mature miRNA sequences or their scrambled counterparts are shown in boldface on grey background in the alignments, or in red in the structure images. The labelling to the right of aligned sequences and underneath the structure images denote whether the particular miRNA corresponds to the AJ507799 sequence (shown at the top of each alignment and structure image pair), or is mutated/deleted in the ΔmirBHRF1, ΔmirALL, +mirBART or wt strains. Note that the parental, prototypic 2089 (wt) strain and therefore all mutant strains inherit the B95-8 deletion, which affects 16 of the 22 pre-miRNAs encoded in the BART region of AJ507799 (indicated by dashes in the alignments). The B95-8 deletion also truncates and fuses the proximal 3/4ths of the pre-miR-BART5 region to sequences located within the *LF1* open reading frame (shown in italics in the alignments on page 2 of the Supporting Figure S1). As shown in the structure predictions for the wt / B95-8 strain (page 9, left structure image in the lower panel), these fused pre-miRNA sequences are unable to form a hairpin and therefore cannot produce a mature miR-BART5 species, even though its coding sequence is left intact. The coding region was nevertheless scrambled in ΔmirBHRF1 as well as ΔmirALL (see bottom alignment and right structure image on pages 2 and 9, respectively). In contrast, in +mirBART the capacity to produce miR-BART5 as well as the 16 deleted pre-miRNAs was restored by insertion of an expression cassette (see main text for details). The region encoding the mature ebv-miR-BART4* was not scrambled in ΔmirBHRF1 or ΔmirALL because this miRNA had not been discovered at the time of mutant design. However, the mutation of the mature ebv-miR-BART4 sequences efficiently destroys the pre-miRNA hairpin structure (see alignment on page 1 and structure prediction on page 8 of Supporting Figure S1) and therefore also ablates expression of ebv-miR-BART4*.
(0.75 MB PDF)

Figure S2 Statistical cell cycle analysis of LCLs infected with miRNA mutant EBVs. LCLs infected with the different miRNA mutant EBVs were cultivated for up to five months and analyzed for their cell cycle distributions as in Figure 4B. The fractions of cells in S phase [%] from four independent experiments were analyzed by the paired t test (two-tailed). The significance values were calculated and shown above the boxes and whiskers (10 to 90% percentiles). Means (+) are indicated. LCLs infected with ΔmiBHRF1 and ΔmirALL mutant EBVs showed a slight reduction of cells in S phase when compared to prototype 2089 and +mirBART EBV-infected cells, which was mostly statistically significant (p≤0.05) suggesting a possible role of EBV's BHRF1 miRNAs in controlling cell proliferation in established LCLs.
(0.03 MB PDF)

Figure S3 Quantitative RT-PCR analysis of BHRF1 mRNA transcripts in prototype 2089 or ΔmirBHRF1 EBV-infected cells. (A) Relative expression of BHRF1 mRNA levels in established LCLs infected with the prototype 2089 (wt) or ΔmirBHRF1 EBV compared to a reference LCL infected with a *BZLF1* knockout EBV (ΔZ) [41], [51] that does not support lytic transcription of *BHRF1*. cDNA synthesis was performed from total RNA after DNase I treatment as described previously [41]. The quantitative PCR reaction was performed with the following conditions: 95°C for 10 min for initial denaturation followed by 45 cycles at 95°C for 1sec, 60°C for 10sec, and 72°C for 7sec. Primer sequences are listed in Supporting Table S3. The obtained values were normalized to the cellular transcript of cytochrome c as an internal reference and expressed relative to the normalized value of the LCL line ΔZ, which served as a control and reference. The arbitrary value of 1 was assigned to this LCL, which had been established with a lytic-cycle deficient *BZLF1*-knockout mutant EBV [51]. The error bars represent the standard deviation of the means of cells from five different donors. (B) Relative expression levels of the BHRF1 transcript in primary B cells at day 5 p.i. infected with prototype 2089 or ΔmirBHRF1 EBV. Primary human B cells isolated from adenoids were infected at a concentration of 4.5×10^5^ per ml with an MOI of 0.2. Data represent the means and standard deviations of three independent experiments. Uninfected cells are indicated (Cont).
(0.12 MB PDF)

Figure S4 Early expression levels of two BHRF1 and four BART miRNAs in primary B cells infected with the prototype 2089 EBV or selected miRNA mutant EBVs. Primary human B cells of three donors isolated from adenoids (4.5{mulitply} 10^5^ per ml) were infected with prototype 2089 (wt), ΔmirBHRF1 or +mir BART EBVs as indicated with an MOI of 0.2. At day 5 p.i., cells were harvested and two BHRF1 miRNAs, miR-BHRF1-1 (panel A) and miR-BHRF1-2-3p (panel B) and four BART miRNAs, miR-BART1-5p (panel C), miR-BART2-5p (panel D), miR-BART8-5p (panel E), and miR-BART 22 (panel F) were quantified by stem-loop PCR assays as described in Materials and Methods. In primary cells early after infection miR-BHRF1-1 and miR-BHRF1-2-3p are expressed at about four- and twofold higher levels, respectively, as compared to established LCLs (Figure 2). Similarly, BART miRNAs are expressed higher in freshly infected cells as compared to their established LCLs but the extent varies. In case of miR-BART22 levels were increased about ninefold, but miR-BART8-5p was only 1.5fold higher expressed than in LCLs established with +mirBART EBV, for example (compare Figure 2E and Supporting Figure S4E). JM LCL is a spontaneous LCL infected with an uncharacterized field strain of EBV encoding 44 viral miRNAs; ΔmirBHRF1 EBV served as a negative control. Data were assessed and evaluated as in Figure 2.
(0.16 MB PDF)

Table S1 Primers used to amplify the *galK* targeting cassette.
(0.05 MB DOC)

Table S2 DNA oligonucleotides for quantitative stem-loop PCR assays.
(0.06 MB DOC)

Table S3 PCR primer pairs for detection of cDNAs.
(0.04 MB DOC)
