# Supplementary material for: Micro RNAs of Epstein-Barr Virus Promote Cell Cycle Progression and Prevent Apoptosis of Primary Human B Cells
Source: PLoS Pathog. 2010 Aug 19;6(8):e1001063. doi: 10.1371/journal.ppat.1001063 (PMC2924374; doi:10.1371/journal.ppat.1001063)
Supplement: Figure S1 — Alignments and predicted structures of mutant miRNAs. This multi-page figure shows alignments (pages 1 to 6) and predicted secondary structure images (pages 7 to 13) of the 25 pre-miRNAs encoded by EBV field strains (represented by the EBV GenBank entry AJ507799) and the corresponding sequences in the mutant strains (ΔmirBHRF1, ΔmirALL, +mirBART) as well as the prototypic parental 2089 EBV strain (wt), which is a molecular clone of the EBV B95.8 genome. Secondary structures were predicted using the Vienna RNAfold package [53] and are indicated by bracket notation above and below the aligned sequences. Regions that encode mature miRNA sequences or their scrambled counterparts are shown in boldface on grey background in the alignments, or in red in the structure images. The labelling to the right of aligned sequences and underneath the structure images denote whether the particular miRNA corresponds to the AJ507799 sequence (shown at the top of each alignment and structure image pair), or is mutated/deleted in the ΔmirBHRF1, ΔmirALL, +mirBART or wt strains. Note that the parental, prototypic 2089 (wt) strain and therefore all mutant strains inherit the B95-8 deletion, which affects 16 of the 22 pre-miRNAs encoded in the BART region of AJ507799 (indicated by dashes in the alignments). The B95-8 deletion also truncates and fuses the proximal 3/4ths of the pre-miR-BART5 region to sequences located within the LF1 open reading frame (shown in italics in the alignments on page 2 of the Supporting Figure S1). As shown in the structure predictions for the wt / B95-8 strain (page 9, left structure image in the lower panel), these fused pre-miRNA sequences are unable to form a hairpin and therefore cannot produce a mature miR-BART5 species, even though its coding sequence is left intact. The coding region was nevertheless scrambled in ΔmirBHRF1 as well as ΔmirALL (see bottom alignment and right structure image on pages 2 and 9, respectively). In contrast, in +mirBART the capaci [file ppat.1001063.s001.pdf]

```
ebv-miR-BHRF1-1
                       ebv-miR-BHRF1-1
          (((.((((.(((((((..((((.(((((.((((.............)))).))))).))))..)))))))....)))))))
AJ507799  GGCUCCUUAUUAACCUGAUCAGCCCCGGAGUUGCCUGUUUCAUCACUAACCCCGGGCCUGAAGAGGUUGACAAGAAGGGUC      wt, +miRBART
          |||||||||| |   | | | |        |||||||||||||||||||||||||||||||||||||||||||||||||||
          GGCUCCUUAUGAUAAUAACCCGGGUGCCCCUUGCCUGUUUCAUCACUAACCCCGGGCCUGAAGAGGUUGACAAGAAGGGUC      ΔmirBHRF1, ΔmirAll
          (((.((((.((.......((((((.((.....))..(((........)))))))))(((....)))....))..)))))))
```

```
ebv-miR-BHRF1-2
                         ebv-miR-BHRF1-2*                    ebv-miR-BHRF1-2
          ((((..(((((((((.(((((((((((.(((((((...........))))))).))))))))))).)))))))))..))))
AJ507799  GGCCCCCACUUUUAAAUUCUGUUGCAGCAGAUAGCUGAUACCCAAUGUUAUCUUUUGCGGCAGAAAUUGAAAGUGCUGGCC      wt, +miRBART
          |||||||||||||   |          || |    ||||||||||||| |   ||   |   |  |||  |||||||||||
          GGCCCCCACUUUUCGGUAGGUGAUAUCCAAACUAUUGAUACCCAAUGUGAAACUUCUGGAUCGUGAUUAUAAGUGCUGGCC      ΔmirBHRF1, ΔmirAll
          ((((..(((((.(((((((............)))))))...(((............)))...........)))))..))))
```

```
ebv-miR-BHRF1-3
                         ebv-miR-BHRF1-3
          ((((((.((((.((((((..((((((.((((((...(((.........)))))))))))))))..)))))).)))).))).)))
AJ507799  CCUGUGGUGUUCUAACGGGAAGUGUGUAAGCACACACGUAAUUUGCAAGCGGUGCUUCACGCUCUUCGUUAAAAUAACACAAGG  wt, +miRBART
          ||||||||||||| | |  |||       | |  ||||||||||||||||||||||||||||||||||||||||||||||||||
          CCUGUGGUGUUCUGAAGAUAAGGUCCACGGAAUGCACGUAAUUUGCAAGCGGUGCUUCACGCUCUUCGUUAAAAUAACACAAGG  ΔmirBHRF1, ΔmirAll
         ((((((((((((((..(((...)))..))))))))............(((((.((.....))...))))).......))).)))
```

```
ebv-miR-BART3
                       ebv-miR-BART3*                       ebv-miR-BART3
         (((((((((..((((.(((((((((.(((((((............))))))).)))))).))).))))..)))))))))
AJ507799 CCUUUGGUGGAACCUAGUGUUAGUGUUGUGCUGUAAAUAAGUGUCCAGCGCACCACUAGUCACCAGGUGUCACCGGAGG        wt, +miRBART
         ||||||||||| |||||| |  ||  |||  ||||||||||||||||| |   |       | | |  | |||||||||
         CCUUUGGUGGAGCCUAGUUUCGGUUGUGUAAUGUAAAUAAGUGUCCAGUGUGUCCACCACAAACUGCCGGCACCGGAGG        ΔmirBHRF1, ΔmirAll
         (((((((((.((((.......))))....................((((.(((.....))).))))....)))))))))
```

```
ebv-miR-BART4
                         ebv-miR-BART4                          ebv-miR-BART4*
         ((...((((((((.(((((.((((((((((((((((............)))))))))).)))).)).))))).))))))))..))
AJ507799 UAACAUUUGGUGGGACCUGAUGCUGCUGGUGUGCUGUAAAUAAGUGCCUAGCACAUCACGUAGGCACCAGGUGUCACCAGGGCUA  wt, +miRBART
         ||||||||||||| |     |  ||     ||  |||||||||||||||||||||||||||||||||||||||||||||||||||
         UAACAUUUGGUGGCAGAGCCUUUUGGGUCGGUCGUGUAAAUAAGUGCCUAGCACAUCACGUAGGCACCAGGUGUCACCAGGGCUA  ΔmirBHRF1, ΔmirAll
         .....((((((((((((.((....)).))..............((((((.((.......))))))))....))))))))))....
```

```
ebv-miR-BART1
                        ebv-miR-BART1-5p                      ebv-miR-BART1-3p
         ((((.((.(((...((((((((((((..((((((((.........)))))))))))).))))))))...))).))))))
AJ507799 CCCGUGGGGGGUCUUAGUGGAAGUGACGUGCUGUGAAUACAGGUCCAUAGCACCGCUAUCCACUAUGUCUCGCCCGGG          wt, +miRBART
         ||||||||||||    |     | |  | |||     ||||||||||||| |        |  ||         |||||||||
         CCCGUGGGGGGUGGGACCUUAUGGUAUGUGGAUCGAAUACAGGUCCACAAUUAGAUUCACCCUCCCUGGUCGCCCGGG          ΔmirBHRF1, ΔmirAll
         .(((.(((((((((.(((....))).((((((((.......))))))))).......))))))))).))).........
```

```
ebv-miR-BART15
                                                      ebv-miR-BART15
         .(((...(((..(((((((((.((((((.(((..((((.....)))))))))))))).))))))))....))).))).
AJ507799 UGUGCCGCUUGGAGGGAAACAUGACCACCUGAAGUCUGUUAACCAGGUCAGUGGUUUUGUUUCCUUGAUAGAGACACA          wt, +mirBART
         ||||||||||||||||||||||||||||||||||||||||||||||      | ||||||||        ||||||||||
         UGUGCCGCUUGGAGGGAAACAUGACCACCUGAAGUCUGUUAACCAGUCGCUUUGUUUUGUUGUGACAGUAGAGACACA          ΔmirBHRF1, ΔmirAll
         .(((...(((...((..((((.(((........)))))))..)).(((((...........)))))....))).))).
```

```
ebv-miR-BART5
                                                            ┌──────────────► b95-8 del
                      ebv-miR-BART5                    ebv-miR-BART5*
         (((.(((....((((((((..(((.((((((((................))))))))).))))))))))))...))).)))
AJ507799 GUGGCACCUCAAGGUGAAUAUAGCUGCCCAUCGACGUAUCGCUGGAAACCGGUGGGCCGCUGUUCACCUAAAGUGACGC        +mirBART
         ||||||||||||||||||||||||||||||||||||||||||||||||||||||||||    |         |  |  |
         GUGGCACCUCAAGGUGAAUAUAGCUGCCCAUCGACGUAUCGCUGGAAACCGGUGGGCCAGGGCCUGGCCGCACGGGCCA        wt
         ....((((....)))).........((((((((..((.((....)).)))))))))).((((.....)))).........

                                                            ┌──────────────► b95-8 del
                      ebv-miR-BART5                    ebv-miR-BART5*
         (((.(((....((((((((..(((.((((((((................))))))))).))))))))))))...))).)))
AJ507799 GUGGCACCUCAAGGUGAAUAUAGCUGCCCAUCGACGUAUCGCUGGAAACCGGUGGGCCGCUGUUCACCUAAAGUGACGC        +mirBART
         |||||||||      | ||    ||  |      |||||||||||||||||||||||||||    |         |  |  |
         GUGGCACCUUUCAAUCAACGGAGUCGAAGUCCGACGUAUCGCUGGAAACCGGUGGGCCAGGGCCUGGCCGCACGGGCCA        ΔmirBHRF1, ΔmirAll
         ....((((....)))).........((((((((..((.((....)).)))))))))).((((.....)))).........
```

```
ebv-miR-BART16
                          ebv-miR-BART16
         (((.(((((.((...((((((..(((((.(((.(((((..........))))).))))))))))))))...)).))))).)))
AJ507799 CAGGUGUGGAAUUUAGAUAGAGUGGGUGUGUGCUCUUGUUUAAUUACACCAAGAUCACCACCCUCUAUCCAUAUCCCACAAUUG   +mirBART

         -------------------------------------------------------------------------------------   wt, ΔmirBHRF1, ΔmirAll
         -------------------------------------------------------------------------------------
```

ebv-miR-BART17

ebv-miR-BART17-5p ebv-miR-BART17-3p

(((...(((((((.((((((((((((((((((............)))))))))))).)))))).))))).))..)))

AJ507799 GUGGCACCC**UAAGAGGACGCAGGCAUACAAG**GUUAUUACCCAGUCCU**UGUAUGCCUGGUGUCCCCUUAGU**GGGACGC +mirBART

--- wt, ΔmirBHRF1, ΔmirAll

----

ebv-miR-BART6

ebv-miR-BART6-5p ebv-miR-BART6-3p

((((....(((((((((((((((((.((((...((.((((......)))).))..)))).)))))))))))))))))...))))

AJ507799 CUUGUUGGUACUU**UAAGGUUGGUCCAAUCCAUAGG**CUUUUUUUGUGAAAACC**CGGGGAUCGGACUAGCCUUAGA**GUAACUCAAG +mirBART

-- wt, ΔmirBHRF1, ΔmirAll

----

ebv-miR-BART21

ebv-miR-BART21-5p ebv-miR-BART21-3p

((.(((((((((((..((((((((...(((((((.(((.......))).)))))))...))))))))..))))))).)))).))

AJ507799 GUAUGGGCUGGGUAU**UCACUAGUGAAGGCAACUAAC**ACAGUUAGACGUG**CUAGUUGUGCCCACUGGUGUUU**AUCCGGUCCCAAAU +mirBART

-- wt, ΔmirBHRF1, ΔmirAll

----

ebv-miR-BART18

ebv-miR-BART18-5p ebv-miR-BART18-3p

((((((..((((...(((((((.((((((.((((((.((....)).)))))).)))))).)))))))....))))..))))))

AJ507799 CGGGUGUCCUGGC**UCAAGUUCGCACUUCCUAUACA**GUGUUAAAGCCUUG**UAUCGGAAGUUUGGGCUUCGUC**CCAGUGUACUCG +mirBART

-- wt, ΔmirBHRF1, ΔmirAll

----

ebv-miR-BART7

ebv-miR-BART7* ebv-miR-BART7

((((((..(((.((((((((((.((((((((..((............))..)))))))).)).)))))))).)))..)))).))

AJ507799 CCAGUGUCCUGAU**CCUGGACCUUGACUAUGAAACA**AUUCUAAAAAAAUG**CAUCAUAGUCCAGUGUCCAGGG**ACAGUGCACUCGG +mirBART

-- wt, ΔmirBHRF1, ΔmirAll

----

ebv-miR-BART8

ebv-miR-BART8 ebv-miR-BART8*

(((((.(((((.((((((..((.((((((((((((..........)))).)))))))).))..)))))).))))).)))))

AJ507799 UGGGUUCACUGAU**UACGGUUUCCUAGAUUGUACAG**AUGAACUAGAACU**GUCACAAUCUAUGGGGUCGUAGA**CAGUGUGCUUA +mirBART

-- wt, ΔmirBHRF1, ΔmirAll

---

ebv-miR-BART9

ebv-miR-BART9* ebv-miR-BART9

(((.((((((..(((.((((((((((.((...((((...........))))..)).)))).)))))).)))..))))))...)))

AJ507799 AGCUGUUGUUUG**UACUGGACCCUGAAUUGGAAAC**AGUAACUUGGAUUCUG**UAACACUUCAUGGGUCCCGUAGU**GACAACUAUGCU +mirBART

--- wt, ΔmirBHRF1, ΔmirAll

---

ebv-miR-BART22

ebv-miR-BART22

((((..(((((((.(((((((((.(((..(((((((.........)))).)))..))).))))))))).))))))).))))

AJ507799 GCGGUUGUCACAGGUGCUAGACCCUGGAGUUGAACCAGUACCACUCGG**UUACAAAGUCAUGGUCUAGUAGU**UGUGACCCUGC +mirBART

-- wt, ΔmirBHRF1, ΔmirAll

---

ebv-miR-BART10

ebv-miR-BART10* ebv-miR-BART10

((((((..(((.(((((.((((.(((((.((((((...........)))))).))))).)))).))))).)))..))))))

AJ507799 GGAGUGUCCCGGG**GCCACCUCUUUGGUUCUGUACA**UAUUUUGUUAUUG**UACAUAACCAUGGAGUUGGCUGU**GGUGCACUCC +mirBART

--- wt, ΔmirBHRF1, ΔmirAll

---

ebv-miR-BART11

ebv-miR-BART11-5p ebv-miR-BART11-3p

((((.((((((((.(((.((((((((((.((..(((((.......)))))...)).)))))))))).))).))))))))...))))

AJ507799 GGCUUCUGUUGGG**UCAGACAGUUUGGUGCGCUAGUUG**UGUGCUUAGCAGCA**ACGCACACCAGGCUGACUGCC**UUAGCAGUGUGGCC +mirBART

--- wt, ΔmirBHRF1, ΔmirAll

---

ebv-miR-BART12		
	ebv-miR-BART12	
	((((((.(((((.(((((((...((((((.((((((.........)))))).))))))...)))).))).))))).))).)))	
AJ507799	CUGGUGACCUAACACCCGCCCAUCACCACCGGACAGAUUCUGAACUUG**UCCUGUGGUGUUUGGUGUGGUU**UUGGGGUACGCAG	+mirBART
	---	wt, ΔmirBHRF1, ΔmirAll
	---	

ebv-miR-BART19		
	ebv-miR-BART19-5p ebv-miR-BART19-3p	
	(((.(((((((...(((((((.(((((((.((((((............)))))).))))))).)))))))..)))))))...))))))).)))	
AJ507799	CCGUGUCCUGACA**ACAUUCCCCGCAAACAUGACAUG**GGUUAAUUUAAACAUG**UUUUGUUUGCUUGGGAAUGCU**CUUAGGGCCUGG	+mirBART
	---	wt, ΔmirBHRF1, ΔmirAll
	---	

ebv-miR-BART20		
	ebv-miR-BART20-5p ebv-miR-BART20-3p	
	((((((.((.(((((((((.(((((((((.((............)).)))))))))..))))))))).)).))).)))	
AJ507799	AGGGCCUAUUG**UAGCAGGCAUGUCUUCAUUCC**UGCGUACCGAAUGG**CAUGAAGGCACAGCCUGUUACC**AUUGGCACCU	+mirBART
	--	wt, ΔmirBHRF1, ΔmirAll
	--	

ebv-miR-BART13		
	ebv-miR-BART13* ebv-miR-BART13	
	(((((.(((.((.(((.(((.((((...((((((............))))))....))))))).))).)).))).)))))	
AJ507799	GGCACCUCGAU**AACCGGCUCGUGGCUCGUACAG**ACGAUUGUUUGGCUC**UGUAACUUGCCAGGGACGGCUGA**CGAUGUGUU	+mirBART
	--	wt, ΔmirBHRF1, ΔmirAll
	--	

ebv-miR-BART14		
	ebv-miR-BART14* ebv-miR-BART14	
	((((.(((.((((..(((((((((((..(((((((..........)))))))...))))).))))))..))))...)))..))))	
AJ507799	CAGGGGUGGCCGG**UACCCUACGCUGCCGAUUUACA**UAAUAUAAAUUG**UAAAUGCUGCAGUAGUAGGGAU**CUGGACGCGCGACCUG	+mirBART
	---	wt, ΔmirBHRF1, ΔmirAll

```
ebv-miR-BART2
                        ebv-miR-BART2-5p                   ebv-miR-BART2-3p
         (((((..(((..((((((((.((.(((((((((((..........))))))).))))..)).))))))))..)))..)))))
AJ507799 GGACUUCCAGACUAUUUUCUGCAUUCGCCCUUGCGUGUCCAUUGUUGCAAGGAGCGAUUUGGAGAAAAUAAACUGUGAGUUU     wt, +mirBART, ΔmirBHRF1
         ||||||||||||     |            || |||||||||||||||||  |  | | |   |   ||||||||||||||
         GGACUUCCAGACCGAACUUCUUUCCGUGUUUUCCGUGUCCAUUGUUGCAAGAUGAAAAUCGAUUAGGGGAAACUGUGAGUUU     ΔmirAll
         ((((....((((((..........)).)))).....))))..........(((....)))..(((.((....)).)))....
```

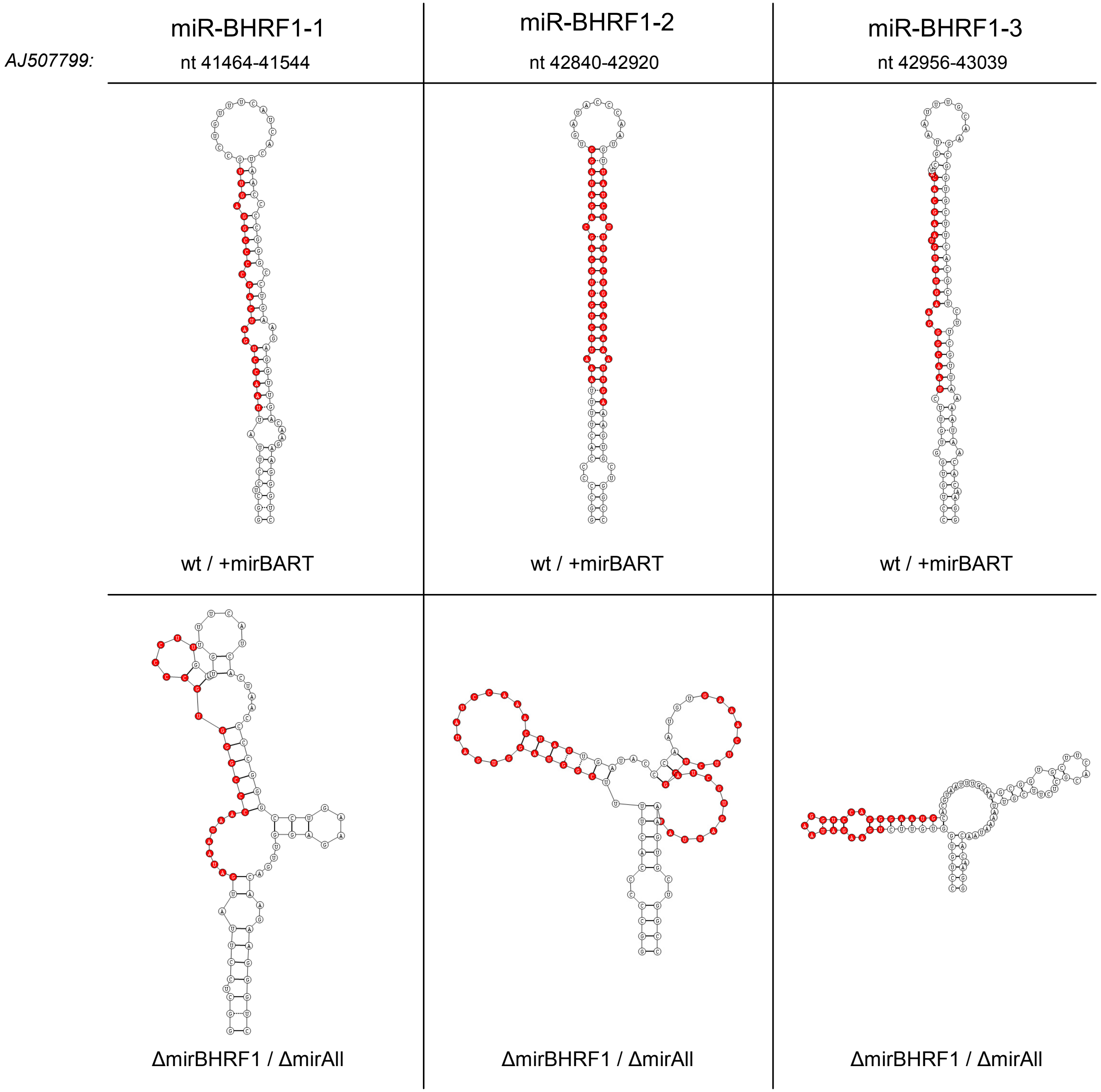
AJ507799:
miR-BHRF1-1
nt 41464-41544
miR-BHRF1-2
nt 42840-42920
miR-BHRF1-3
nt 42956-43039
wt / +mirBART
wt / +mirBART
wt / +mirBART
ΔmirBHRF1 / ΔmirAll
ΔmirBHRF1 / ΔmirAll
ΔmirBHRF1 / ΔmirAll

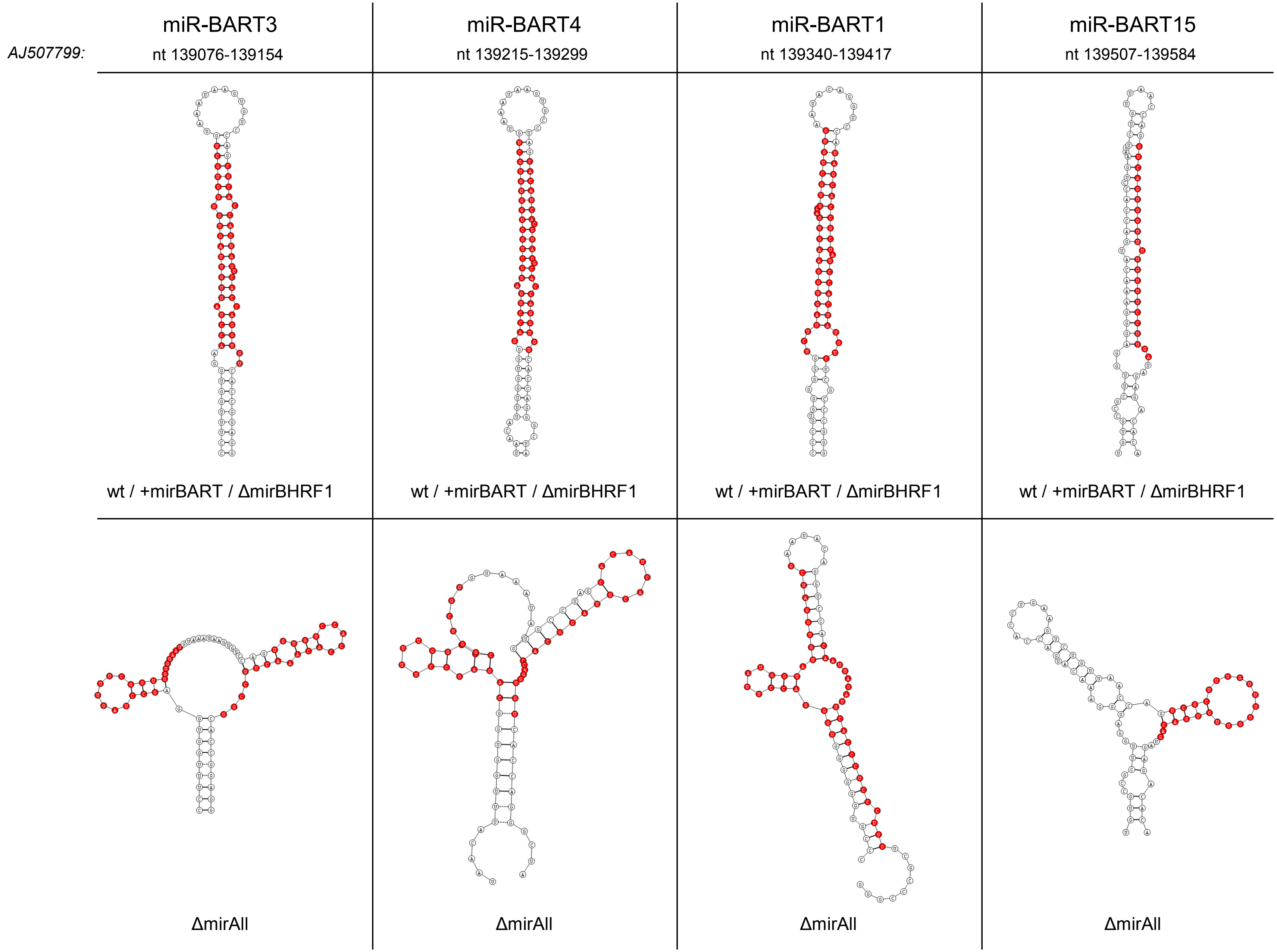
AJ507799:
miR-BART3
nt 139076-139154
miR-BART4
nt 139215-139299
miR-BART1
nt 139340-139417
miR-BART15
nt 139507-139584
wt / +mirBART / ΔmirBHRF1
wt / +mirBART / ΔmirBHRF1
wt / +mirBART / ΔmirBHRF1
wt / +mirBART / ΔmirBHRF1
ΔmirAll
ΔmirAll
ΔmirAll
ΔmirAll

AJ507799:	miR-BART5<br>nt 139666-139744		miR-BART16<br>nt 139783-139866	miR-BART17<br>nt 139906-139982
	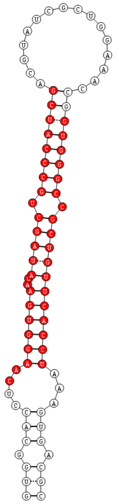<br>+mirBART		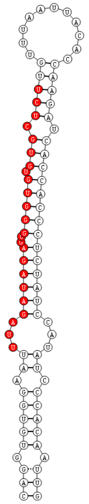<br>+mirBART	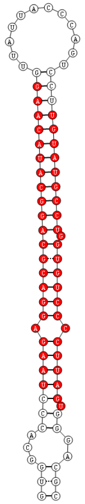<br>+mirBART
	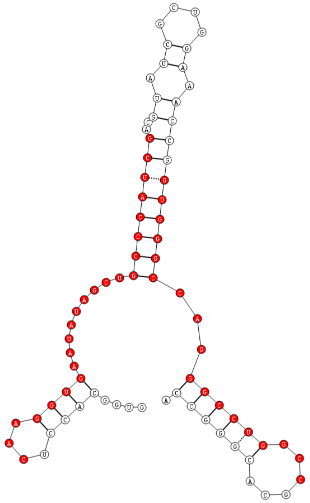<br>wt	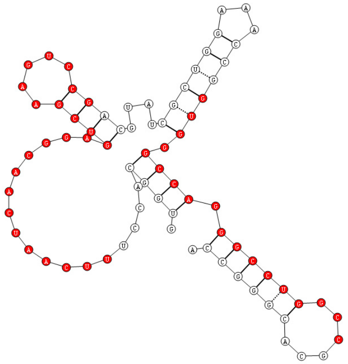<br>ΔmirBHRF1 / ΔmirAll	*deleted*<br>wt / ΔmirBHRF1 / ΔmirAll	*deleted*<br>wt / ΔmirBHRF1 / ΔmirAlll

AJ507799:	miR-BART6<br>nt 140020-104103	miR-BART21<br>nt 145499-145583	miR-BART18<br>nt 145949-145583	miR-BART7<br>nt 146420-146502
	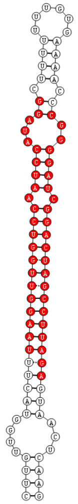<br>+mirBART	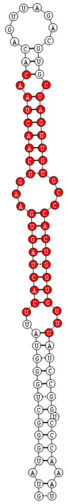<br>+mirBART	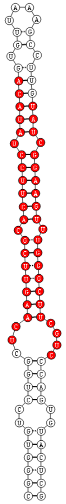<br>+mirBART	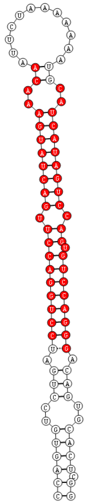<br>+mirBART
	deleted	*deleted*	*deleted*	*deleted*
	wt / ΔmirBHRF1 / ΔmirAll	wt / ΔmirBHRF1 / ΔmirAlll	wt / ΔmirBHRF1 / ΔmirAll	wt / ΔmirBHRF1 / ΔmirAlll

AJ507799:	miR-BART8<br>nt 146759-146840	miR-BART9<br>nt 146947-147031	miR-BART22<br>nt 147155-147236	miR-BART10<br>nt 147308-147388
	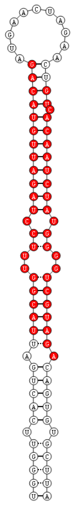<br>+mirBART	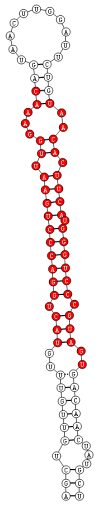<br>+mirBART	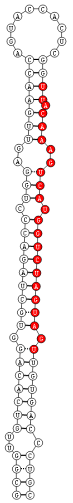<br>+mirBART	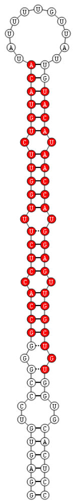<br>+mirBART
	deleted<br>wt / ΔmirBHRF1 / ΔmirAll	*deleted*<br>wt / ΔmirBHRF1 / ΔmirAlll	*deleted*<br>wt / ΔmirBHRF1 / ΔmirAll	*deleted*<br>wt / ΔmirBHRF1 / ΔmirAlll

	miR-BART11	miR-BART12	miR-BART19	miR-BART20
AJ507799:	nt 147524-147609	nt 147888-147970	nt 148202-148286	nt 148328-148405
	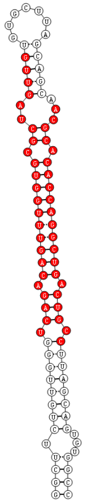 +mirBART	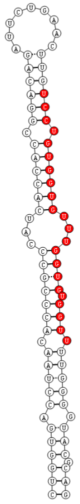 +mirBART	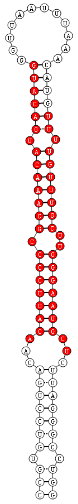 +mirBART	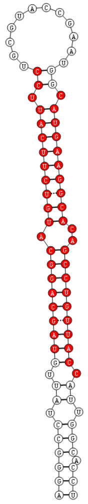 +mirBART
	deleted wt / ΔmirBHRF1 / ΔmirAll	*deleted* wt / ΔmirBHRF1 / ΔmirAlll	*deleted* wt / ΔmirBHRF1 / ΔmirAll	*deleted* wt / ΔmirBHRF1 / ΔmirAlll

AJ507799:	miR-BART13<br>nt 148515-148594	miR-BART14<br>nt 148731-148815	miR-BART2<br>nt 152735-152816
	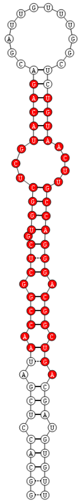<br>+mirBART	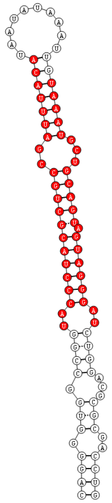<br>+mirBART	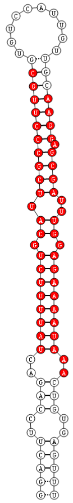<br>wt / +mirBART / ΔmirBHRF1
	deleted<br>wt / ΔmirBHRF1 / ΔmirAll	*deleted*<br>wt / ΔmirBHRF1 / ΔmirAlll	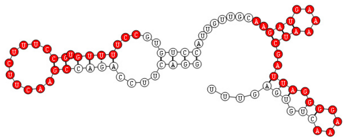<br>ΔmirAll
