# Supplementary figures and images for: Micro RNAs of Epstein-Barr Virus Promote Cell Cycle Progression and Prevent Apoptosis of Primary Human B Cells
Source: PLoS Pathog. 2010 Aug 19;6(8):e1001063. doi: 10.1371/journal.ppat.1001063 (PMC2924374; doi:10.1371/journal.ppat.1001063)

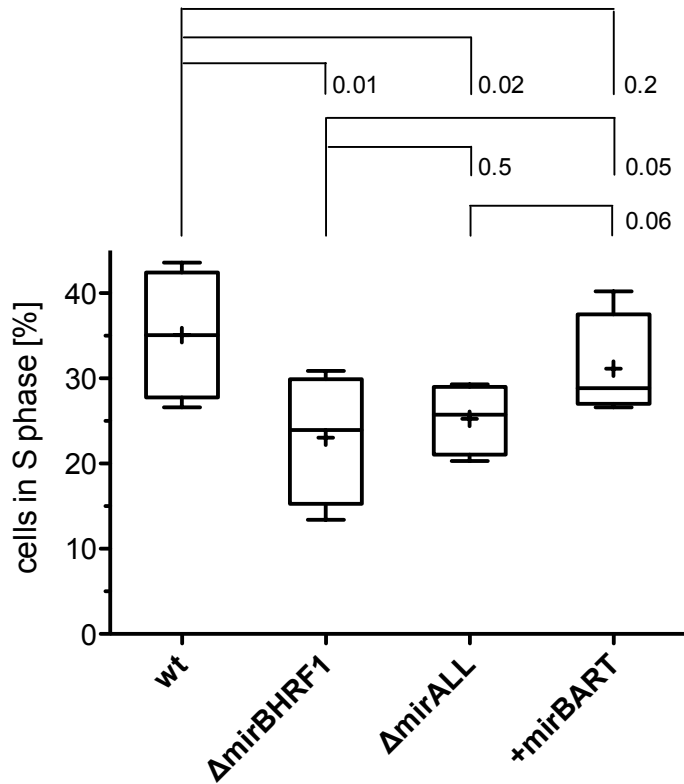

Fig. S2

Supplement: Figure S2 — Statistical cell cycle analysis of LCLs infected with miRNA mutant EBVs. LCLs infected with the different miRNA mutant EBVs were cultivated for up to five months and analyzed for their cell cycle distributions as in Figure 4B. The fractions of cells in S phase [%] from four independent experiments were analyzed by the paired t test (two-tailed). The significance values were calculated and shown above the boxes and whiskers (10 to 90% percentiles). Means (+) are indicated. LCLs infected with ΔmiBHRF1 and ΔmirALL mutant EBVs showed a slight reduction of cells in S phase when compared to prototype 2089 and +mirBART EBV-infected cells, which was mostly statistically significant (p≤0.05) suggesting a possible role of EBV's BHRF1 miRNAs in controlling cell proliferation in established LCLs. (0.03 MB PDF) [file ppat.1001063.s002.pdf]

**A**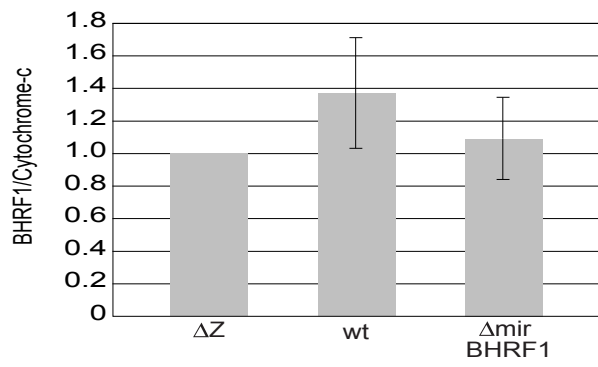**B**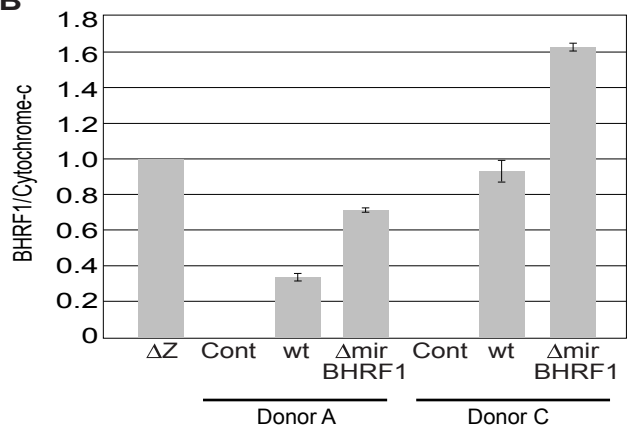

Supplement: Figure S3 — Quantitative RT-PCR analysis of BHRF1 mRNA transcripts in prototype 2089 or ΔmirBHRF1 EBV-infected cells. (A) Relative expression of BHRF1 mRNA levels in established LCLs infected with the prototype 2089 (wt) or ΔmirBHRF1 EBV compared to a reference LCL infected with a BZLF1 knockout EBV (ΔZ) [41], [51] that does not support lytic transcription of BHRF1. cDNA synthesis was performed from total RNA after DNase I treatment as described previously [41]. The quantitative PCR reaction was performed with the following conditions: 95°C for 10 min for initial denaturation followed by 45 cycles at 95°C for 1sec, 60°C for 10sec, and 72°C for 7sec. Primer sequences are listed in Supporting Table S3. The obtained values were normalized to the cellular transcript of cytochrome c as an internal reference and expressed relative to the normalized value of the LCL line ΔZ, which served as a control and reference. The arbitrary value of 1 was assigned to this LCL, which had been established with a lytic-cycle deficient BZLF1-knockout mutant EBV [51]. The error bars represent the standard deviation of the means of cells from five different donors. (B) Relative expression levels of the BHRF1 transcript in primary B cells at day 5 p.i. infected with prototype 2089 or ΔmirBHRF1 EBV. Primary human B cells isolated from adenoids were infected at a concentration of 4.5×10^5^ per ml with an MOI of 0.2. Data represent the means and standard deviations of three independent experiments. Uninfected cells are indicated (Cont). (0.12 MB PDF) [file ppat.1001063.s003.pdf]

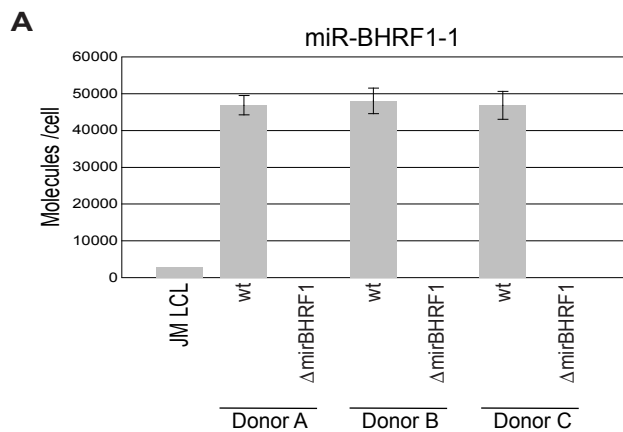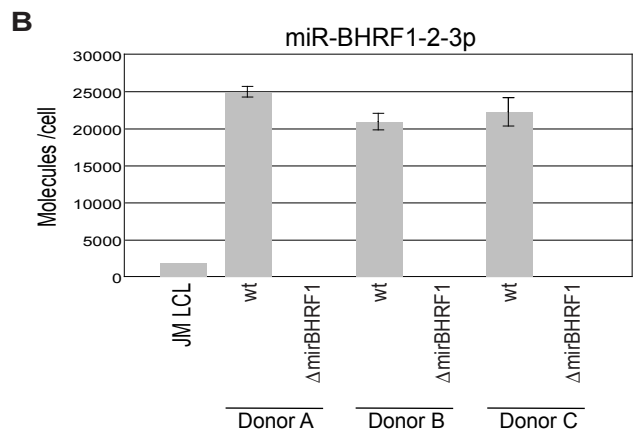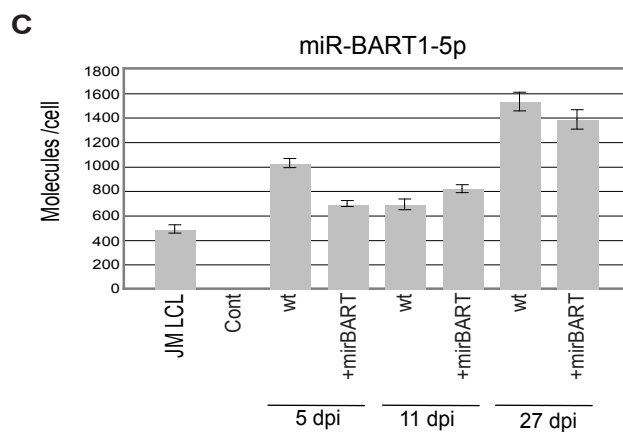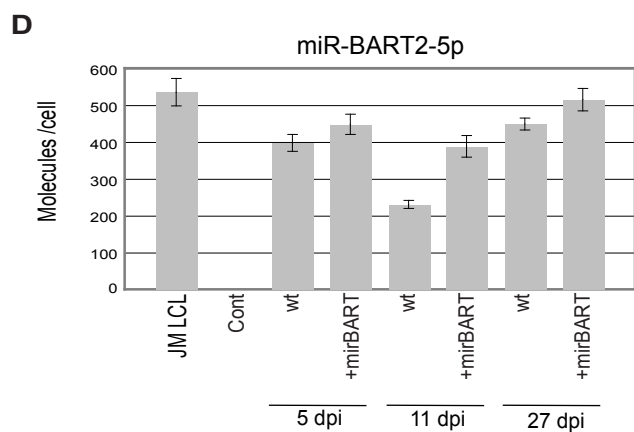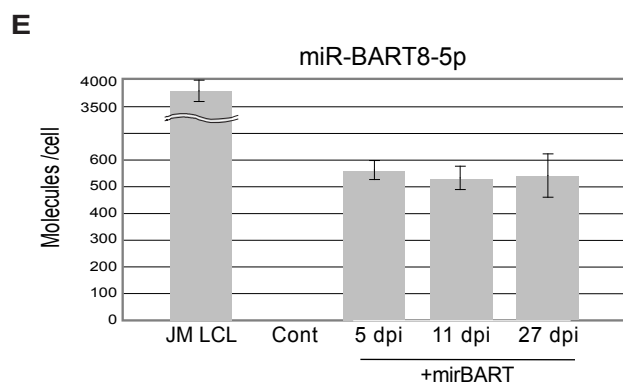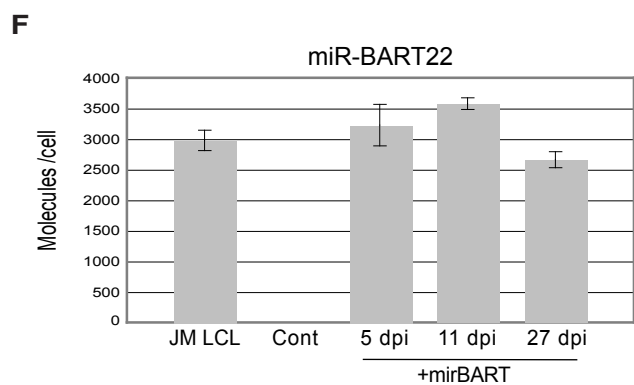

Supplement: Figure S4 — Early expression levels of two BHRF1 and four BART miRNAs in primary B cells infected with the prototype 2089 EBV or selected miRNA mutant EBVs. Primary human B cells of three donors isolated from adenoids (4.5{mulitply} 105 per ml) were infected with prototype 2089 (wt), ΔmirBHRF1 or +mir BART EBVs as indicated with an MOI of 0.2. At day 5 p.i., cells were harvested and two BHRF1 miRNAs, miR-BHRF1-1 (panel A) and miR-BHRF1-2-3p (panel B) and four BART miRNAs, miR-BART1-5p (panel C), miR-BART2-5p (panel D), miR-BART8-5p (panel E), and miR-BART 22 (panel F) were quantified by stem-loop PCR assays as described in Materials and Methods. In primary cells early after infection miR-BHRF1-1 and miR-BHRF1-2-3p are expressed at about four- and twofold higher levels, respectively, as compared to established LCLs (Figure 2). Similarly, BART miRNAs are expressed higher in freshly infected cells as compared to their established LCLs but the extent varies. In case of miR-BART22 levels were increased about ninefold, but miR-BART8-5p was only 1.5fold higher expressed than in LCLs established with +mirBART EBV, for example (compare Figure 2E and Supporting Figure S4E). JM LCL is a spontaneous LCL infected with an uncharacterized field strain of EBV encoding 44 viral miRNAs; ΔmirBHRF1 EBV served as a negative control. Data were assessed and evaluated as in Figure 2. (0.16 MB PDF) [file ppat.1001063.s004.pdf]
