# Supplementary material for: Micro RNAs of Epstein-Barr Virus Promote Cell Cycle Progression and Prevent Apoptosis of Primary Human B Cells
Source: PLoS Pathog. 2010 Aug 19;6(8):e1001063. doi: 10.1371/journal.ppat.1001063 (PMC2924374; doi:10.1371/journal.ppat.1001063)
Supplement: Table S1 — Primers used to amplify the galK targeting cassette. (0.05 MB DOC) [file ppat.1001063.s005.doc]

**Table S1**. Primers used to amplify the *galK* targeting cassette.

| **Targeted miRNAs** | **Primer type** | **Sequence (5’ to 3’)** |
| --- | --- | --- |
| miR-BHRF1-1 | Fw | GATTTTCTATGTGGGGGTGGAAATATGAGCAAGAATAAGGACGGCTCCTTAT  CCTGTTGACAATTAATCATCGGCA |
|  | BW | CTTGACCCTTCTTGTCAACCTCTTCAGGCCCGGGGTTAGTGATGAAACAGGC  TCAGCACTGTCCTGCTCCTT |
| miR-BHRF1-2/2*/3 | Fw | CCGGCCATGGGGGCTTACAAGAATAACATGCCAATGACCCGGCCCCCACTTTT  CCTGTTGACAATTAATCATCGGCA |
|  | BW | TCTTGTCCTTGTGTTATTTTAACGAAGAGCGTGAAGCACCGCTTGCAAATTACGT  TCAGCACTGTCCTGCTCCTT |
| miR-BART3/4/1/15/5 | Fw | AATCCCTGTAAACACACACCACCTAAGAACAAGGCATTGTTAACCTTTGGTGG  CCTGTTGACAATTAATCATCGGCA |
|  | BW | GCATCGATGGCCCGTGCGGCCAGGCCCTGGCCCACCGGTTTCCAGCGATACGT  TCAGCACTGTCCTGCTCCTT |
| miR-BART2 | Fw | CCCAGGCAGTCAGTTATTTTGCATGCCACCTCCCTGCCTGGTGGACTTCCAGAC  CCTGTTGACAATTAATCATCGGCA |
|  | BW | AAAGGCGGTGGGATCATGAAGCCCCCAGGGGAGCGTGGCCCGTGGATCTGTGAAACTCACAG  TCAGCACTGTCCTGCTCCTT |
