# Supplementary material for: Micro RNAs of Epstein-Barr Virus Promote Cell Cycle Progression and Prevent Apoptosis of Primary Human B Cells
Source: PLoS Pathog. 2010 Aug 19;6(8):e1001063. doi: 10.1371/journal.ppat.1001063 (PMC2924374; doi:10.1371/journal.ppat.1001063)
Supplement: Table S2 — DNA oligonucleotides for quantitative stem-loop PCR assays. (0.06 MB DOC) [file ppat.1001063.s006.doc]

**Table S2**. DNA-oligonucleotides for qPCR assay

| **miRNA** | **Primer type** | **Sequence (5’ to 3’)** |
| --- | --- | --- |
| miR-BHRF1-1 | fs^a^ | GTTGGCTCTGGTGCAGGGTCCGAGGTATTCGCACCAGAGCCAACAACTCC |
|  | 5’ | AGAGTAACCTGATCAGCCCC |
| miR-BHRF1-2-3p | fs | GTTGGCTCTGGTGCAGGGTCCGAGGTATTCGCACCAGAGCCAACTCAATT |
|  | 5’ | GGCGTATCTTTTGCGGCAGA |
| miR-BART1-5p | fs | GTTGGCTCTGGTGCAGGGTCCGAGGTATTCGCACCAGAGCCAACCACAGC |
|  | 5’ | CGGTCTTAGTGGAAGTGACGT |
| miR-BART17-5p | fs | GTTGGCTCTGGTGCAGGGTCCGAGGTATTCGCACCAGAGCCAACCTTGTA |
|  | 5’ | GTGTTAAGAGGACGCAGGCA |
| miR-BART22 | fs | GTTGGCTCTGGTGCAGGGTCCGAGGTATTCGCACCAGAGCCAACACTACT |
|  | 5’ | GCGGCTTACAAAGTCATGGTCT |
| miR-BART8-5p | fs | GTTGGCTCTGGTGCAGGGTCCGAGGTATTCGCACCAGAGCCAACCTGTAC |
|  | 5’ | GCCGGTACGGTTTCCTAGATT |
| miR-BART2-5p | fs | GTTGGCTCTGGTGCAGGGTCCGAGGTATTCGCACCAGAGCCAACGCAAGG |
|  | 5’ | GCCGGTATTTTCTGCATTCGC |
| miRNA universal | 3’ | GTGCAGGGTCCGAGGT |

^a^miRNA specific firststrand primer
