# Supplementary material for: Micro RNAs of Epstein-Barr Virus Promote Cell Cycle Progression and Prevent Apoptosis of Primary Human B Cells
Source: PLoS Pathog. 2010 Aug 19;6(8):e1001063. doi: 10.1371/journal.ppat.1001063 (PMC2924374; doi:10.1371/journal.ppat.1001063)
Supplement: Table S3 — PCR primer pairs for detection of cDNAs. (0.04 MB DOC) [file ppat.1001063.s007.doc]

**Table S3**. PCR primers used in this study

| Gene | Primer type | Sequence (5’ to 3’) |
| --- | --- | --- |
| BZLF1 | 5’ | AAGCCACCCGATTCTTGTATCG |
|  | 3’ | CAGCAGCAGCAGTGGTGTTTG |
| BHRF1 | 5’ | CTGGATGGTTGGATTCATCA |
|  | 3’ | TGGATCCAGGAATGTTGTCTT |
| Cytochrome-c | 5’ | CCTGGTGGGCGTGTGCTAC |
|  | 3’ | CAATGCTCCGTTGTTGGCAG |
